# Discrimination and calibration performances of non-laboratory-based and laboratory-based cardiovascular risk predictions: a systematic review

**DOI:** 10.1136/openhrt-2024-003147

**Published:** 2025-02-10

**Authors:** Yihun Mulugeta Alemu, Sisay Mulugeta Alemu, Nasser Bagheri, Kinley Wangdi, Dan Chateau

**Affiliations:** 1 National Centre for Epidemiology and Population Health, College of Health and Medicine, Australian National University, Canberra, Australian Capital Territory, Australia; 2 Department of Epidemiology and Biostatistics, School of Public Health, Bahir Dar University College of Medical and Health Sciences, Bahir Dar, Amhara, Ethiopia; 3 Department of Health Science, University of Groningen, Groningen, The Netherlands; 4 Health Research Institute, University of Canberra, Canberra, Australian Capital Territory, Australia; 5 HEAL Global Research Center, Research Institute, University of Canberra, Canberra, Australian Capital Territory, Australia

**Keywords:** Metabolic Diseases, Coronary Artery Disease, Acute Coronary Syndrome, Epidemiology, Health Services

## Abstract

**Background and objective:**

This review compares non-laboratory-based and laboratory-based cardiovascular disease (CVD) risk prediction equations in populations targeted for primary prevention.

**Design:**

Systematic review.

**Methods:**

We searched five databases until 12 March 2024 and used prediction study risk of bias assessment tool to assess bias. Data on hazard ratios (HRs), discrimination (paired c-statistics) and calibration were extracted. Differences in c-statistics and HRs were analysed. Protocol: PROSPERO (CRD42021291936).

**Results:**

Nine studies (1 238 562 participants, 46 cohorts) identified six unique CVD risk equations. Laboratory predictors (eg, cholesterol and diabetes) had strong HRs, while body mass index in non-laboratory models showed limited effect. Median c-statistics were 0.74 for both models (IQR: lab 0.77–0.72; non-lab 0.76–0.70), with a median absolute difference of 0.01. Calibration measures between laboratory-based and non-laboratory-based equations were similar, although non-calibrated equations often overestimated risk.

**Conclusion:**

The discrimination and calibration measures between laboratory-based and non-laboratory-based models show minimal differences, demonstrating the insensitivity of c-statistics and calibration metrics to the inclusion of additional predictors. However, in most reviewed studies, the HRs for these additional predictors were substantial, significantly altering predicted risk, particularly for individuals with higher or lower levels of these predictors compared with the average.

## Introduction

 Cardiovascular disease (CVD) is the major cause of mortality and morbidity globally.^1^ More than half a billion people globally were affected by CVD in 2021, resulting in 20.5 million deaths, representing nearly a third of all deaths worldwide.^2 3^ The majority of CVD deaths occur in low- and middle-income countries (LMICs).^4^ The mortality trends in high-income countries demonstrate that deaths attributed to CVD are mostly preventable.^5^

CVD risk prediction equations, which account for various risk factors of CVD, are frequently used in primary care settings to identify individuals who have a higher risk of developing CVD and who would likely benefit from preventive measures.^6 7^

While numerous CVD risk prediction equations have been developed and used for estimating CVD risk and guiding treatment strategies,^8^,^10^ their application in LMICs is limited due to the high cost of blood lipid-level measurements, which many equations rely on as inputs.^11^ There are also CVD risk equations that use alternative non-laboratory measures, such as body mass index (BMI).^12 13^ Although developed for use in LMIC settings, most non-laboratory CVD risk equations have been developed using data from non-LMIC populations.^14 15^

While previous studies have compared non-laboratory and laboratory equations in various settings,^16^,^18^ a comprehensive review comparing measures of discrimination and calibration between laboratory-based and non-laboratory-based risk equations, as well as evaluating the effect of hazard ratios (HRs) for additional predictors in predicting CVD risk, was lacking. There was a need for a systematic comparison between laboratory-based and non-laboratory-based equations, focusing on discrimination, calibration measures and the HRs of additional predictors, which is important for assessing the relative predictive performances of the competing CVD risk equations across diverse populations to ensure their generalisability.^19 20^

This review aims to assess and compare the performance of laboratory and non-laboratory CVD risk equations in populations different from those in which they were originally developed. The review has relevance to LMICs, aiming to identify effective risk assessment and intervention strategies with limited facilities, thus contributing to global efforts to reduce the burden of CVD.

## Methods

### Scope of review

This review addressed three key questions: (1) to compare the HRs of additional predictors in CVD risk prediction; (2) to identify externally validated non-laboratory-based and laboratory-based CVD equations from the same cohort and outline the reported model performance measures (discrimination and calibration) and (3) to analyse overall differences in model performance measures between laboratory-based and non-laboratory equations.

### Search strategy and selection criteria

This systematic review protocol was registered under PROSPERO (CRD42021291936). Our reporting adheres to the Preferred Reporting Items for Systematic Review and Meta-Analyses (PRISMA) guidelines^21^ and the Meta-Analysis of Observational Studies in Epidemiology^22^ (online supplemental appendix B). We systematically searched for studies published in five databases: PubMed, Scopus, Web of Science, ProQuest Dissertations and Theses Global and Google Scholar. The search began with the start of the review on 4 April 2023 and continued until 12 March 2024. We used combinations of search terms related to “laboratory-based”, “non-laboratory-based” and “cardiovascular risk scores” (detailed search terms and strategies provided in online supplemental appendix A). Searches were deliberately broad to encompass all relevant studies. Additionally, reference lists of included studies were screened to find further relevant studies. The included studies were published between 2002 and 2021.

### Inclusion and exclusion criteria

Articles were included in this study if they met the following criteria: (a) compared laboratory-based and non-laboratory-based CVD risk prediction equations within the same study population; (b) were undertaken in a study population that differed to the population that the risk equation was derived in (ie, external validation study); (c) equations that were not recalibrated for the target population and (d) papers published in the English language. We focused on externally validated equations since external validation is essential for assessing the reproducibility and generalisability of a prediction model in diverse sets of new populations.^23^ We only examined equations externally validated in populations where recalibration was not undertaken prior to validation. Recalibration aims to align the risks predicted by the equation with the risks observed in the target population. Recalibration approaches would, therefore, mask any differences in calibration between laboratory and non-laboratory equations if included in the analysis.^24 25^ Articles were excluded if: (a) the study included people with existing CVD or a history of CVD at baseline; (b) non-English literature; (c) conference abstracts and (d) case reports.

### Screening, quality assessment and data extraction

Titles and abstracts were independently reviewed by two authors (YMA and SMA). Full-text reviews were independently undertaken by the same authors. Research articles that fulfilled the inclusion criteria were evaluated for quality assessment by two authors (YMA and SMA). Disagreements on article selection and quality assessment were resolved through discussion. Two authors (YMA and SMA) extracted data using a predefined data extraction form. We extracted study-level data on age, sex, year of data collection, number of study participants, types of CVD risk equations and outcome measures (HRs, paired c-statistics, CIs, calibration χ^2^, calibration plots, calibration slopes (CSs), observed and expected ratio). A preliminary version of the Cochrane Prediction Study Risk of Bias Assessment Tool for cohort studies was used to assess the level of bias by evaluating selected parameters, including participant selection, predictors, outcome, sample size, participant flow and analysis^26 27^(online supplemental appendix D).

### Data synthesis and analysis

This systematic review compared CVD risk equations using laboratory-based models with cholesterol and non-laboratory-based models using variables, such as BMI, synthesising adjusted HRs and regression coefficients from multivariable models. For example, the WHO-2019 and Ueda Globorisk equations extracted HRs for risk factors, such as age, systolic blood pressure, diabetes, smoking, total cholesterol and BMI. Similarly, the D’Agostino Framingham and Persian Atherosclerotic CVD equations incorporated cholesterol and diabetes in laboratory-based models, and BMI, waist–hip ratio (WHR) or diabetes history in non-laboratory models. The D’Agostino Framingham equations used log-transformed continuous variables to improve discrimination, calibration and minimise the effects of extreme observations. The estimated regression coefficients for the D’Agostino Framingham and Persian Atherosclerotic CVD equations were presented alongside the HRs. We included HRs because they directly quantify the impact of each predictor on individual CVD risk, providing clinically relevant insights beyond c-statistics. HRs are particularly useful for comparing laboratory-based and non-laboratory-based models, as large differences in HRs can significantly influence risk predictions, thereby enhancing model sensitivity and clinical utility.^28 29^ Calibration measures are also less sensitive to changes in predictor inclusion and may be less effective in capturing the influence of additional predictors.^24^ Discrimination, a measure of how well a risk prediction equation distinguishes between those with and without the disease, was assessed by extracting data on c-statistics from the included studies. C-statistics typically range from 0.5 (random concordance) to 1 (perfect concordance).^30^,^32^ As a standard, c-statistics<0.70 indicate inadequate discrimination, between 0.70 and 0.80 are considered acceptable and between 0.80 and 0.90 are considered excellent.^6 33^

We computed c-statistics differences between laboratory-based and non-laboratory-based equations by subtracting the non-laboratory-based c-statistics from the laboratory-based, which were compared within the same population. Forest plots were used to present the c-statistics and c-statistics differences. We calculated the absolute differences for each pair of c-statistics values; then, we computed the median absolute difference in c-statistics across all studies. Differences in c-statistics (changes in c-statistics) are classified into four categories. Large is used when the difference is 0.1 or greater; moderate for 0.05 to 0.1; small for 0.025 to 0.05 and very small for less than 0.025. Because c-statistics range from 0.5 to 1.0, the 0.1 cut-off point for large was set because it represents 20% of the possible range.^34^

We compared calibration between laboratory and non-laboratory CVD risk equations using four calibration measures (where available in the publications). First, we examined two χ^2^ metrics: the Hosmer–Lemeshow χ^2^ and Greenwood Nam-D’Agostino statistics, considering a significance level of p value<0.05 or a χ^2^ statistic exceeding 20 as indicative of a significant lack (poor calibration).^35 36^ Second, we examined how the population’s CVD risk was divided into risk deciles and plotted the predicted event rates against the observed.^16^ Third, we evaluated the ratio of expected to observed outcomes, or their probabilities, with a ratio close to 1 indicating effective model calibration.^37^ Finally, we considered a model’s CS, where a slope below 1 suggests overfitting, while slopes above 1 suggest underfitting. A slope near 1 indicates good calibration in the validation dataset.^38^ All analyses were performed using R (version 4.3.0).

## Results

Overall, nine studies met the inclusion criteria,^1116 17 39^,^43^ with 1 238 562 study participants, from 46 cohorts included (online supplemental appendix C).^1116 18 39 41 44^,^65^ The cohorts consisted of 5 LMICs, along with 19 upper-middle-income and high-income countries, making a total of 24 countries analysed (online supplemental table 1). The median years of enrollment of cohorts used for external validation of the included studies ranged from 1961 to 2008 (a study may use more than one cohort for external validation). Studies excluded from the full-text stage are detailed in online supplemental appendix E. In general, the studies included were found to have a minimal risk of bias.

**Table 1:** HRs and performance of the WHO-2019 and ueda globorisk CVD risk equations

WHO-2019 risk equation (based on ERFC data)		
Outcomes: fatal or non-fatal MI or CHD death		
Women		
Risk factor	Laboratory-basedHR (95% CI)	Non-laboratory-basedHR (95% CI)
Age at baseline per 5 years	1.66 (1.60 to 1.73)	1.69 (1.62 to 1.75)
SBP per 20 mm Hg	1.38 (1.34 to 1.42)	1.40 (1.36 to 1.45)
History of diabetes	2.91 (2.59 to 3.27)	NA
Current smoking	2.83 (2.61 to 3.08)	2.94 (2.71 to 3.20)
Total cholesterol per 1 mmol/L	1.23 (1.20 to 1.26)	NA
BMI per 5 kg/m^2^	NA	1.14 (1.10 to 1.18)
C-statistics (95% CI)	0.7570 (0.7492 to 0.7648)	0.7382 (0.7301 to 0.7463)
Men		
Age at baseline per 5 years	1.43 (1.40 to 1.46)	1.44 (1.41 to 1.48)
SBP per 20 mm Hg	1.30 (1.28 to 1.33)	1.31 (1.28 to 1.33)
History of diabetes	1.89 (1.75 to 2.04)	NA
Current smoking	1.76 (1.68 to 1.85)	1.81 (1.73 to 1.90)
Total cholesterol per 1 mmol/L	1.26 (1.24 to 1.28)	NA
BMI per 5 kg/m^2^	NA	1.18 (1.15 to 1.22)
C-statistics (95% CI)	0.6890 (0.6839 to 0.6941)	0.6660 (0.6610 to 0.6720)
Outcomes: fatal or non-fatal stroke		
Women		
Age at baseline per 5 years	1.70 (1.64 to 1.76)	1.69 (1.63 to 1.75)
SBP per 20 mm Hg	1.51 (1.46 to 1.56)	1.54 (1.49 to 1.60)
History of diabetes	2.35 (2.06 to 2.70)	NA
Current smoking	2.11 (1.92 to 2.31)	2.10 (1.91 to 2.31)
Total cholesterol per 1 mmol/L	1.05 (0.95 to 1.16)	NA
BMI per 5 kg/m^2^	NA	1.02 (0.98 to 1.06)
C-statistics (95% CI)	0.7440 (0.736 to 0.753)	0.7367 (0.7282 to 0.7453)
Men		
Age at baseline per 5 years	1.64 (1.58 to 1.70)	1.63 (1.57 to 1.69)
SBP per 20 mm Hg	1.56 (1.52 to 1.61)	1.58 (1.53 to 1.63)
History of diabetes	1.88 (1.68 to 2.11)	NA
Current smoking	1.65 (1.53 to 1.77)	1.65 (1.53 to 1.78)
Total cholesterol per 1 mmol/L	1.03 (1.00 to 1.06)	NA
BMI per 5 kg/m^2^	NA	1.08 (1.03 to 1.13)
C-statistics (95% CI)	0.7265 (0.7186 to 0.7345)	0.7233 (0.7152 to 0.7315)
Ueda globorisk extension		
Women and men combined		
Systolic blood pressure per 10 mm Hg	1.18 (1.16 to 1.19)	1.18 (1.17 to 1.20)
Diabetes	1.88 (1.71 to 2.06)	NA
Female with diabetes	1.50 (1.29 to 1.75)	NA
Smoker	1.55 (1.44 to 1.66)	1.52 (1.42 to 1.64)
Female smoker	1.38 (1.21 to 1.59)	1.42 (1.24 to 1.63)
Total cholesterol per 1 mmol/L	1.19 (1.16 to 1.22)	NA
BMI per 5 kg/m^2^	NA	1.14 (1.11 to 1.17)
C-statistics (95% CI)	0.71 (0.70 to 0.72)	0.69 (0.68 to 0.70)

BMIbody mass indexCHDcoronary heart diseaseCVDcardiovascular diseaseERFCemerging risk factors collaborationHRhazard ratioMImyocardial infarctionNAnot applicableSBPsystolic blood pressure

For included studies, six non-laboratory-based risk equations along with their corresponding laboratory-based risk equations were reported: WHO-2019; D’Agostino Framingham; Ueda Globorisk extension; European Prospective Investigation into Cancer and Nutrition (EPIC); INTERHEART and Persian Atherosclerotic CVD Risk Stratification (PARS). The laboratory measurements included in the risk equations varied. WHO-2019 and the Ueda Globorisk extension laboratory equations included both total cholesterol and diabetes as laboratory predictors,^11 18^ while D'Agostino Framingham, PARS and EPIC used total cholesterol, HDL cholesterol and diabetes,^15 16 66^ and the INTERHEART equation used apolipoproteins.^67^ (online supplemental table 2) provides a summary of the variables included in each equation, the study populations and predicted outcome events. Non-laboratory-based versions of WHO-2019, the Ueda Globorisk extension and D’ Agostino Framingham used BMI as a substitute for laboratory measures,^11 15 18^ while EPIC, SPARS and INTERHEART used WHR and/or non-clinical factors, such as diet.^16 65 67^

**Table 2:** HR and performance of D’Agostino Framingham and Persian atherosclerotic CVD risk equations

D’Agostino Framingham risk equations (Framingham cohort)				
Women				
Variables	Laboratory-based		Non-laboratory-based	
	β*	HR (95% CI)	β*	HR (95% CI)
Log of age	2.32888	10.27 (5.65 to 18.64)	2.72107	15.20 (8.59 to 26.87)
Log of total cholesterol	1.20904	3.35 (2.00 to 5.62)	NA	NA
Log of HDL cholesterol	−0.70833	0.49 (0.35 to 0.69)	NA	NA
Log of SBP if not treated	2.76157	15.82 (7.86 to 31.87)	2.81291	16.66 (8.27 to 33.54)
Log of SBP if treated	2.82263	16.82 (8.46 to 33.46)	2.88267	17.86 (8.97 to 35.57)
Smoking	0.52873	1.70 (1.40 to 2.06)	0.61868	1.86 (1.53 to 2.25)
Diabetes	0.69154	2.00 (1.49 to 2.67)	0.77763	2.18 (1.63 to 2.91)
BMI	NA	NA	0.51125	1.67 (0.98 to 2.85)
C-statistics (95% CI)	0.793 (0.772 to 0.814)		0.785 (0.764 to 0.806)	
Men				
Log of age	3.06117	21.35 (14.03 to 32.48)	3.11296	22.49 (14.80 to 34.16)
Log of total cholesterol	1.12370	3.08 (2.05 to 4.62)	NA	NA
Log of HDL cholesterol	−0.93263	0.39 (0.30 to 0.52)	NA	NA
Log of SBP if not treated	1.93303	6.91 (3.91 to 12.20)	1.85508	6.39 (3.61 to 11.33)
Log of SBP if treated	1.99881	7.38 (4.22 to 12.92)	1.92672	6.87 (3.90 to 12.08)
Smoking	0.65451	1.92 (1.65 to 2.24)	0.70953	2.03 (1.75 to 2.37)
Diabetes	0.57367	1.78 (1.43 to 2.20)	0.53160	1.70 (1.37 to 2.11)
BMI	NA	NA	0.79277	2.21 (1.25 to 3.91)
C-statistics (95% CI)	0.763 (0.746 to 0.780)		0.749 (0.731 to 0.767)	
PARS equation in Tehran lipid and glucose cohort				
Sex (men and women)				
Age	0.04592	1.047 (1.04 to 1.054)	0.04494	1.046 (1.039 to 1.053)
Male	0.71764	2.050 (1.748 to 2.403)	0.76677	2.153 (1.801 to 2.573)
Total cholesterol (mg/dL)				
<150	–	1 (Reference)	NA	NA
150–200	0.00956	1.010 (0.627 to 1.625)		
200–250	0.41855	1.520 (0.956 to 2.417)		
250–300	0.55737	1.746 (1.087 to 2.804)		
>300	0.95743	2.605 (1.571 to 4.321)		
SBP (mm Hg)				
<120	–	1 (Reference)		1 (Reference)
120–139	0.15221	1.164 (0.967 to 1.402)	0.21399	1.239 (1.029 to 1.490)
140–159	0.60783	1.836 (1.493 to 2.259)	0.70242	2.019 (1.643 to 2.481)
>=160	0.74208	2.100 (1.643 to 2.684)	0.84719	2.333 (1.825 to 2.982)
Diabetes	0.79142	2.207 (1.899 to 2.564)	NA	NA
High WHR	0.31902	1.376 (1.154 to 1.640)	NA	NA
Family history of CVD	0.38066	1.463 (1.240 to 1.727)	NA	NA
Smoking	0.49554	1.641 (1.341 to 2.010)	0.47038	1.601 (1.308 to 1.959)
WHR				
1	NA	NA	–	1 (Reference)
2			0.35549	1.427 (1.188 to 1.713)
3			0.55846	1.748 (1.399 to 2.184)
4			0.69904	2.012 (1.577 to 2.567)
History of diabetes	NA	NA	0.88382	2.420 (2.064 to 2.837)
C-statistics (95% CI)	0.78 (0.76 to 0.79)		0.77 (0.75 to 0.78)	

WHR was classified into four categories: 1, 2, 3 and 4, with the following cut-off points: in females, <0.85, 0.85–0.90, 0.90–0.95 and ≥0.95; in males, <1.00, 1.00–1.05, 1.05–1.10 and ≥1.10.

* estimated regression coefficient.

BMIbody mass indexCVDcardiovascular diseaseHDLhigh-density lipoproteinHRhazard ratioNAnot applicablePARSPersian Atherosclerotic Cardiovascular Disease Risk StratificationSBPsystolic blood pressureWHRwaist–hip ratio

From the included studies, we extracted data on 64 HRs, 30 paired c-statistics (30 laboratory-based and 30 non-laboratory-based),^1116 17 39^,^43^ 5 paired calibration χ^2^,^41 43 65^ 22 paired calibration plots,^11 16 17 40 41 43 65^ 7 paired CSs^17^ and 1 paired observed–expected ratio^43^ (online supplemental table 3).

### Comparison of HRs and performance between non-laboratory-based and laboratory-based CVD risk equations

For most risk equations examined, HRs for laboratory-based measures (eg, diabetes and cholesterol) were higher than those for non-laboratory-based measures, such as BMI. It is important to note that these HRs are unstandardised, meaning the variables (eg, binary for diabetes, continuous for cholesterol in mg/dL and BMI in kg/m²) are measured in their original units, they are not expressed on a common scale. For example, in the WHO-2019 CVD risk study using data from the emerging risk factors collaboration in women, the HRs for diabetes (HR 2.91, 95% CI 2.59 to 3.27) and total cholesterol (HR 1.23, 95% CI 1.20 to 1.26) in the laboratory-based model were higher than the HR for BMI (HR 1.14, 95% CI 1.10 to 1.18), which was included in the non-laboratory-based models. Both models included smoking, age and systolic blood pressure. The laboratory-based model had a c-statistics of 0.757 (95% CI 0.749 to 0.765), compared with 0.738 (95% CI 0.730 to 0.746) for the non-laboratory-based model.

In the Ueda Globorisk extension risk equation, the laboratory-based model included diabetes (HR 1.88, 95% CI 1.71 to 2.06) and total cholesterol (HR 1.19, 95% CI 1.16 to 1.22), while the non-laboratory-based model included BMI (HR 1.14, 95% CI 1.11 to 1.17). The c-statistics for the laboratory-based model was 0.71 (95% CI 0.70 to 0.72) compared with 0.69 (95% CI 0.68 to 0.70) for the non-laboratory-based model (table 1). In the Framingham cohort dataset in women, the laboratory-based model included total cholesterol (HR 3.35, 95% CI 2.00 to 5.62) and HDL cholesterol (HR 0.49, 95% CI 0.35 to 0.69), while the non-laboratory-based model substituted BMI (HR 1.67, 95% CI 0.98 to 2.85). The c-statistics for the laboratory-based model was 0.793 (95% CI 0.772 to 0.814), and for the non-laboratory-based model, 0.785 (95% CI 0.764 to 0.806). In the ICS cohort, the PARS laboratory-based model included the additional predictor of total cholesterol>300 mg/dL (HR 1.73, 95% CI 1.17 to 2.56), with c-statistics of approximately 0.73 for both the non-laboratory-based model (95% CI 0.71 to 0.74) and the laboratory-based model (95% CI 0.71 to 0.75) (table 2).

### Comparison of discrimination measures

Overall, most CVD risk equations showed good discrimination, with 26 of the 30 c-statistics pairs being>0.7. The median external validation c-statistics in the laboratory-based equations was 0.74 (IQR, 0.77–0.72), and the median external validation c-statistics in the non-laboratory-based was also 0.74 (IQR, 0.76–0.70) online supplemental figure 1. There was little difference in c-statistics between laboratory-based and non-laboratory-based. The median absolute difference in the c-statistics between laboratory-based and non-laboratory-based was 0.01 (IQR, 0.01–0.00) (online supplemental figure 2). Within individual studies, 26 out of the 30 c-statistics differences were very small (differences in c-statistics<0.025) and 4 c-statistics differences were considered small (differences in c-statistics were 0.025–0.05); 3 of which were observed in the INTERHEART equation and 1 in the Globorisk equation.

### Comparison of calibration measures between laboratory-based and non-laboratory-based equations

Overall, calibration measures of the externally validated risk equations suggested both the laboratory-based and non-laboratory-based D’Agostino Framingham risk prediction equations,^68^ overestimating the observed risk in Australia,^39^ Germany^16^ and the UAE population^40^ and the Atherosclerosis Risk in Communities from four distinct US provinces: Forsyth County, North Carolina; Jackson, Mississippi; suburbs of Minneapolis, Minnesota and Washington County, Maryland. Both the laboratory-based and non-laboratory-based EPIC-Potsdam equations marginally overestimated risk in the highest decile of predicted risk. The observed and expected ratios for the EPIC-Potsdam equation were O: E=1.05 (95% CI 0.97 to 1.13) for the non-laboratory-based and O: E=1.11 (95% CI 1.03 to 1.20) for the laboratory-based in the Heidelberg, German populations, respectively.^16^

According to the INTERHEART equation CSs, in South America, the equation showed a degree of overfitting in the non-laboratory-based version, with a CS of 0.87 (95% CI 0.77 to 0.98). Similarly, in China, both the non-laboratory-based and laboratory-based equations showed overfitting, with CS of 0.81 (0.71–0.91) and 0.88 (0.78–0.98), respectively. The non-laboratory-based equation showed overfitting in Africa with a CS of 0.75 (0.36–1.15). Overfitting patterns persist in South Asia and North America/Europe, with CS values of 0.75 (0.65–0.86) and 0.77 (0.68–0.87), respectively, for the non-laboratory-based equation. Conversely, the INTERHEART laboratory-based equation underfits in Middle Eastern populations, with a CS of 1.41 (1.18–1.63).^17^

External validations of both non-laboratory-based and laboratory-based PARS/SPARS equations showed an overestimation of observed event rates in the Iranian population (χ^2^ p value of less than 0.001).^42^ Similarly, external validations of both non-laboratory-based and laboratory-based WHO-2019 equations identified an overestimation of CVD risk in Chinese populations (χ^2^ p value of less than 0.001).^45^ None of the included studies assessed the calibration of the Globorisk extension equation^11^ in an external validation study.

## Discussion

This study is the first systematic review that summarises and compares the HRs, discrimination and calibration performance of non-laboratory-based and laboratory-based CVD risk prediction equations; the evaluated equations were externally validated in primary prevention populations. In most CVD equations, the inclusion of predictors, such as cholesterol and diabetes, demonstrated stronger HRs than BMI. Discrimination performance was similar between the laboratory and non-laboratory among the six CVD risk equations reviewed, with absolute differences in c-statistics of less than 0.05. While c-statistics differences may overlook correlations between exposures and outcomes in competing models, net reclassification improvement (NRI) addresses this; however, most studies in the review did not use NRI, emphasising the need for its inclusion in future model evaluations.^69^ The majority of the CVD risk equations, both laboratory-based and non-laboratory-based, showed discrimination>0.7. However, few studies were well calibrated to external validation datasets or populations.

In the systematic review, laboratory-based predictors, such as total cholesterol and diabetes, demonstrated stronger HRs, indicating better risk stratification, whereas non-laboratory measures, such as BMI, had weaker HRs and a more limited influence on risk stratification. In most risk equations, laboratory-based models show similar c-statistics or slight improvements over non-laboratory-based models. Adding new variables may not significantly affect c-statistics, which often remain similar despite added predictors. However, incorporating predictors, such as diabetes and cholesterol, enhances risk stratification by yielding significantly higher HRs, thereby improving the identification of individuals at higher CVD risk and supporting clinical decision-making.^70^,^73^

Similar to our findings, previous studies have identified that most CVD equations in use had a c-statistics greater than 0.7 discrimination performance in external validation studies.^68 74 75^ In our review, 26 out of 30 pairs of discrimination measures had a c-statistics of 0.70 or higher.

In this review, many of the CVD risk equations showed poor calibration during external validation, emphasising the need for recalibration. Models with poor calibration may either underestimate or overestimate the outcome of interest.^76^ Factors, such as limited derivation dataset diversity, changes in population characteristics and shifts in underlying CVD risk, contribute to lower calibration of CVD risk equations.^77^ However, in our review, we found no evidence that calibration differed between laboratory-based and non-laboratory-based CVD risk equations when assessed in the same population.

Recalibration is a statistical adjustment aligning predicted and observed risk in the target population.^78^ Recalibration is necessary when applying CVD risk equations to populations with different underlying risk levels.^9 35 79^ Recalibration methods, such as intercept and slope adjustment, piecewise recalibration and transformation of predictors, can be employed to ensure that predicted risks closely match observed outcomes in predictive models, thereby enhancing the accuracy and reliability of CVD risk assessment.^80 81^

C-statistics are relatively insensitive to the inclusion of new predictors in a model, even when those predictors are statistically and clinically significant.^71^ Evaluating calibration in risk equations is challenging, as a slope of 1 suggests good calibration, but poor calibration can still occur if the intercept is overlooked, emphasising the need for a comprehensive assessment that includes both slope and intercept, along with other calibration metrics.^82^ The Hosmer–Lemeshow test assesses calibration by comparing observed and expected event rates in subgroups, but it has limitations, such as arbitrary groupings and reduced power in small datasets.^83^ Calibration plots compare predicted and observed risks, but smoothing techniques and arbitrary groupings (eg, by deciles) can affect accuracy, especially in small datasets. These plots may also overlook important or subtle differences across risk groups, so using them with other metrics is recommended for a more comprehensive model evaluation.^24 83 84^ Like other calibration measures, the observed-to-expected outcome ratio has a limitation, as it only assesses the average agreement between predicted and observed risks, without considering variations across different risk levels.^85 86^

This review identified five LMICs, including Bangladesh, India, Iran, Pakistan and Zimbabwe, out of a total of 24 countries analysed.^11 17 18 42^ Given that the majority of studies were conducted in high-income countries, there is a need for further research in LMICs comparing laboratory and non-laboratory equations. Most CVD risk equations globally have been derived or validated using cohorts from high-income settings. However, prospective cohort studies in LMICs are scarce, particularly where non-laboratory-based CVD risk equations are needed due to limited access to laboratory facilities, such as lipid testing. This review focuses on externally validated CVD risk equations to assess model reproducibility and generalisability. Future research that compares the predictive performance of non-laboratory risk equations with laboratory-based models that were not externally validated is warranted.^1639^,^41^

### Strengths and limitations

This is the first review of the discrimination and calibration performance of paired non-laboratory-based and laboratory-based CVD risk prediction equations, reflecting both comprehensiveness and broad scope, while also incorporating the HRs of additional predictors evaluated in competing models to further enhance comparison. This review evaluated six externally validated CVD risk equations in primary prevention populations across 24 countries, providing a comparative analysis that offers valuable insights into the performance of various prediction models. The inclusion of studies from diverse global populations enhances the applicability and generalisability of the findings. Since c-statistics are influenced by the composition of the study population (eg, age distribution), we only extracted c-statistics from studies where laboratory and non-laboratory CVD risk equations were compared within the same population.^71^ This study focuses on CVD equations that have been externally validated, thereby providing limited insight into the comparison of laboratory and non-laboratory CVD risk equations that have not been externally validated. Many CVD risk equations are recalibrated before application in new populations to align predicted risks with observed outcomes. While this recalibration improves model performance, it may obscure differences in the original calibration of the equations. Therefore, our review focused on non-recalibrated equations to evaluate their baseline predictive abilities between laboratory-based and non-laboratory-based models. However, in practice, many CVD risk equations are recalibrated for the specific population they are intended for, which may result in better calibration than what was observed in this study.^77^ Most of the studies in this review were conducted in high-income countries, while non-laboratory CVD risk equations are most applicable in LMICs, emphasising the urgent need for prospective cohort studies in LMICs to assess their CVD risk profiles and inform the derivation and external validation of context-specific equations.

## Conclusion

The discrimination and calibration measures between laboratory-based and non-laboratory-based models show minimal differences, demonstrating the insensitivity of c-statistics and calibration metrics to the inclusion of additional predictors. However, in most reviewed studies, the HRs for these additional predictors were substantial, significantly altering predicted risk, particularly for individuals with higher or lower levels of these predictors compared with the average.

## supplementary material

10.1136/openhrt-2024-003147 online supplemental file 1

10.1136/openhrt-2024-003147 online supplemental file 2

10.1136/openhrt-2024-003147 online supplemental file 3

10.1136/openhrt-2024-003147 online supplemental file 4

10.1136/openhrt-2024-003147 online supplemental file 5

10.1136/openhrt-2024-003147 online supplemental file 6

## Data Availability

Data are available on reasonable request.
