# Supplementary figures and images for: Discrimination and calibration performances of non-laboratory-based and laboratory-based cardiovascular risk predictions: a systematic review
Source: Open Heart. 2025 Feb 10;12(1):e003147. doi: 10.1136/openhrt-2024-003147 (PMC11815431; doi:10.1136/openhrt-2024-003147)

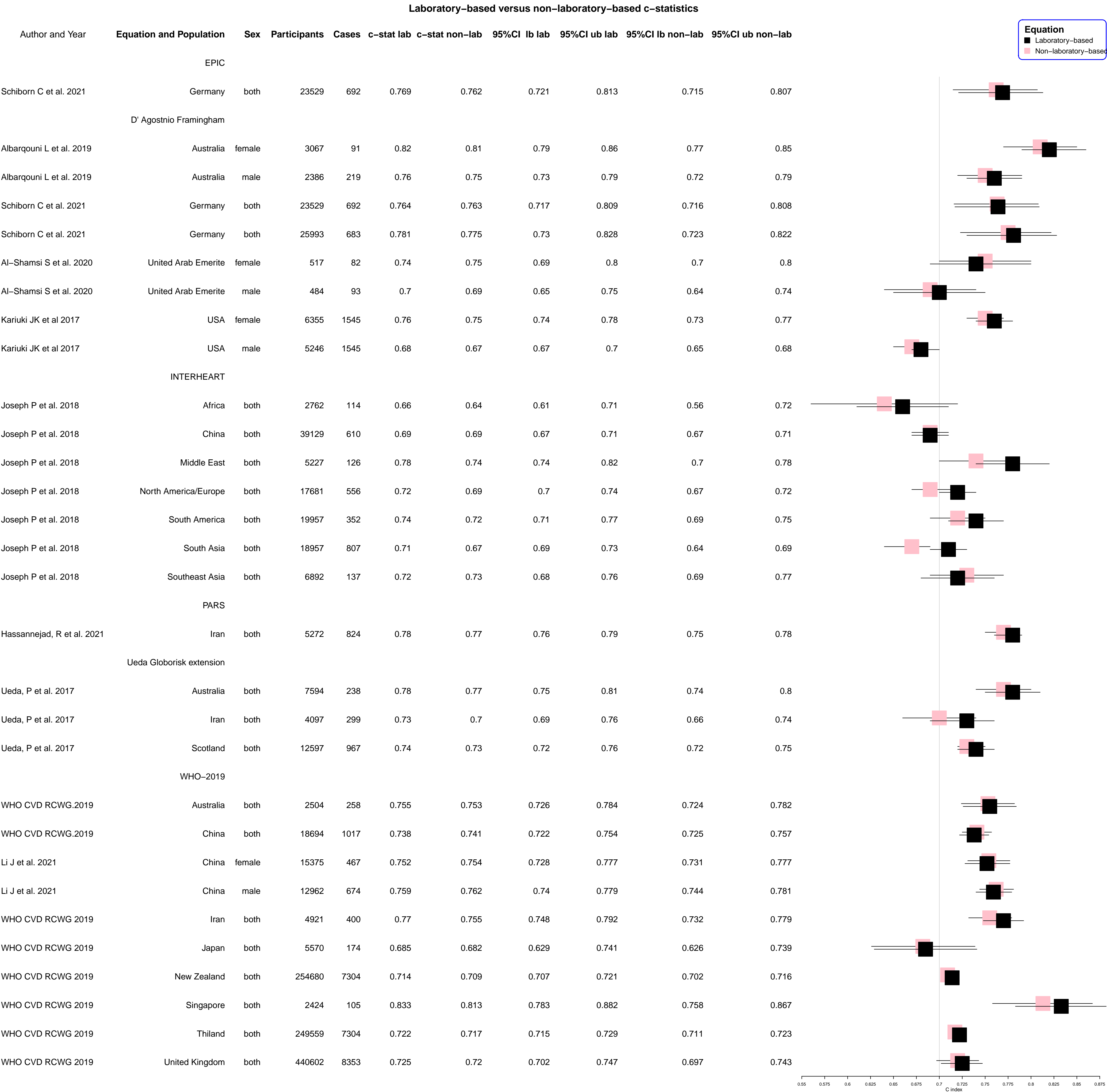

Supplement: online supplemental file 1 [file openhrt-12-1-s001.pdf]

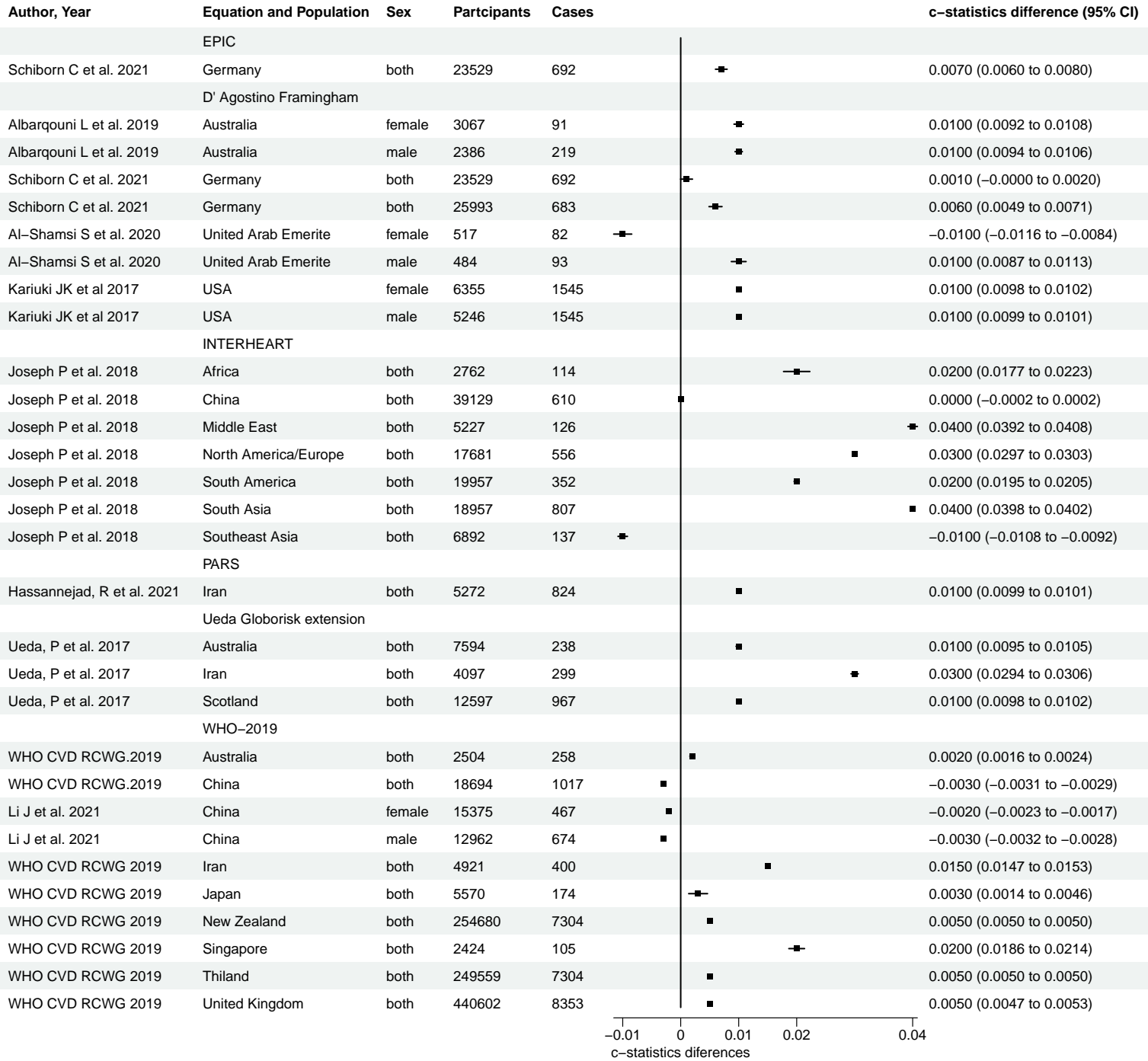

Supplement: online supplemental file 2 [file openhrt-12-1-s002.pdf]
