# Supplementary material for: Discrimination and calibration performances of non-laboratory-based and laboratory-based cardiovascular risk predictions: a systematic review
Source: Open Heart. 2025 Feb 10;12(1):e003147. doi: 10.1136/openhrt-2024-003147 (PMC11815431; doi:10.1136/openhrt-2024-003147)
Supplement: online supplemental file 3 [file openhrt-12-1-s003.docx]

Supplementary: Table 1: Characteristics of Included Studies

| Author(year) | Study design | Externally  validated equation | Source population and data collection period for external validation | Reported model performance measures |
| --- | --- | --- | --- | --- |
| Schiborn C et al. 2021 ^1^. | Prospective cohort | EPIC-Potsdam clinical and non-clinical ^1^. | Germany-Heidelberg (1994 to 2012) ^1 2^. | c-statistics, calibration plots, and expected-to-observed ratios |
|  | Prospective cohort | D'Agostino Framingham  laboratory-based and non-laboratory-based ^3^. | Germany-Potsdam and Germany-Heidelberg (1994 to 2012) ^1 2^. | c-statistics, calibration plots, and expected-to-observed ratios |
| Albarqouni L et al. 2019 ^4^. | Prospective cohort | D'Agostino Framingham  laboratory-based and non-laboratory-based ^3^. | Australia (1999 to 2011) ^5^. | c-statistics, calibration plots |
| Al-Shamsi S et al. 2022 ^6^. | Retrospective cohort | D'Agostino Framingham  laboratory-based and non-laboratory-based ^3^. | United Arab Emirates  (2008 to 2019) ^6^. | c-statistics and calibration plots |
| Kariuki JK et al 2017 ^7^. | Prospective cohort | D'Agostino Framingham  laboratory-based and non-laboratory-based ^3^ | USA (1987 to 2001) ^7 8^ | c-statistics, calibration chi-square, and calibration plots |
| Joseph P et al (2018) ^9^. | Prospective cohort | INTERHEART laboratory-based and non-laboratory-based ^10^. | Africa, China, the Middle East, North America, Europe, South America, South Asia, and Southeast Asia: a total of 17 cohorts were included 4 in Europe/North America (Canada, Poland, Sweden, and Turkey); 4 in South America (Argentina, Brazil, Chile, and Colombia); 3 in South Asia (India, Pakistan, and Bangladesh); 2 in the Middle East (Iran and the United Arab Emirates); 1 in China; 1 in Southeast Asia (Malaysia); and 2 in Africa (South Africa and Zimbabwe). | c-statistics, calibration slop, and calibration plot |
| Hassannejad R et al (2021) ^11^. | Prospective cohort | PARS ^12^, SPARS ^13^ | Iran (2001 to 2011) | c-statistics , calibration chi2, and calibration plot |
| Ueda, P et al. 2017 ^14^. | Prospective cohort | Ueda Globo risk extension ^14^ . | Australia,  Iran, Scotland,  (1984-2012) | c-statistics |
| WHO 2019 ^15^. | Prospective cohort | WHO 2019 ^15^. | There were 19 cohorts: 2 in Australia, 6 in China, 4 in Japan, 2 in Singapore, 2 in New Zealand, 1 in Iran, 1 in Thailand, and 1 in the UK. The cohorts included those from the Asia Pacific Cohort Studies Collaboration (APCSC) ^16^, the New Zealand primary care-based PREDICT cardiovascular  disease cohort (PREDICTCVD)^17^, the Chinese Multi-Provincial Cohort Study^18^, the Health Checks Ubon Ratchathani Study in Thailand ^19^, the Tehran Lipids and Glucose Study ^20^, and the UK Biobank ^21^. | c-statistics |
| Li J et al. 2021 ^22^. | Prospective cohort | WHO 2019 ^15^. | China-PAR Cohort from 1992 to 2015 ^22^. | c-statistics and calibration chi-square |

EPIC: European Prospective Investigation into Cancer and Nutrition: PARS: Persian Atherosclerotic Cardiovascular Disease Risk Stratification: SPARS: Simplified Non-Laboratory-Based PARS: China-PAR: Prediction for Atherosclerotic Cardiovascular Disease Risk in China.

**References**

1. Schiborn C, Kühn T, Mühlenbruch K, et al. A newly developed and externally validated non-clinical score accurately predicts 10-year cardiovascular disease risk in the general adult population. Sci Rep 2021;11(1):19609. doi: 10.1038/s41598-021-99103-4 [published Online First: 20211004]

2. Bergmann MM, Bussas U, Boeing H. Follow-up procedures in EPIC-Germany--data quality aspects. European Prospective Investigation into Cancer and Nutrition. Ann Nutr Metab 1999;43(4):225-34. doi: 10.1159/000012789

3. D'Agostino RB, Sr., Vasan RS, Pencina MJ, et al. General cardiovascular risk profile for use in primary care: the Framingham Heart Study. Circulation 2008;117(6):743-53. doi: 10.1161/circulationaha.107.699579 [published Online First: 20080122]

4. Albarqouni L, Doust JA, Magliano D, et al. External validation and comparison of four cardiovascular risk prediction models with data from the Australian Diabetes, Obesity, and Lifestyle study. Medical Journal of Australia 2019;210(4):161-67. doi: <https://doi.org/10.5694/mja2.12061>

5. Dunstan DW, Zimmet PZ, Welborn TA, et al. The Australian Diabetes, Obesity and Lifestyle Study (AusDiab)--methods and response rates. Diabetes Res Clin Pract 2002;57(2):119-29. doi: 10.1016/s0168-8227(02)00025-6

6. Al-Shamsi S, Govender RD, King J. External validation and clinical usefulness of three commonly used cardiovascular risk prediction scores in an Emirati population: a retrospective longitudinal cohort study. BMJ Open 2020;10(10):e040680. doi: 10.1136/bmjopen-2020-040680 [published Online First: 20201028]

7. Kariuki JK, Stuart-Shor EM, Leveille SG, et al. Validation of the nonlaboratory-based Framingham cardiovascular disease risk assessment algorithm in the Atherosclerosis Risk in Communities dataset. Journal of Cardiovascular Medicine 2017;18:936–45.

8. The Atherosclerosis Risk in Communities (ARIC) Study: design and objectives. The ARIC investigators. Am J Epidemiol 1989;129(4):687-702.

9. Joseph P, Yusuf S, Lee SF, et al. Prognostic validation of a non-laboratory and a laboratory based cardiovascular disease risk score in multiple regions of the world. Heart 2018;104(7):581-87. doi: 10.1136/heartjnl-2017-311609 [published Online First: 20171024]

10. McGorrian C, Yusuf S, Islam S, et al. Estimating modifiable coronary heart disease risk in multiple regions of the world: the INTERHEART Modifiable Risk Score. Eur Heart J 2011;32(5):581-9. doi: 10.1093/eurheartj/ehq448 [published Online First: 20101222]

11. Hassannejad R, Mansourian M, Marateb H, et al. Developing Non-Laboratory Cardiovascular Risk Assessment Charts and Validating Laboratory and Non-Laboratory-Based Models. Glob Heart 2021;16(1):58. doi: 10.5334/gh.890 [published Online First: 20210902]

12. Sarrafzadegan N, Hassannejad R, Marateb HR, et al. PARS risk charts: A 10-year study of risk assessment for cardiovascular diseases in Eastern Mediterranean Region. PLoS One 2017;12(12):e0189389. doi: 10.1371/journal.pone.0189389

13. Hassannejad R, Mansourian M, Marateb H, et al. Developing Non-Laboratory Cardiovascular Risk Assessment Charts and Validating Laboratory and Non-Laboratory-Based Models. Global Heart 2021;16(1):58-58. doi: 10.5334/gh.890

14. Ueda P, Woodward M, Lu Y, et al. Laboratory-based and office-based risk scores and charts to predict 10-year risk of cardiovascular disease in 182 countries: a pooled analysis of prospective cohorts and health surveys. Lancet Diabetes Endocrinol 2017;5(3):196-213. doi: 10.1016/s2213-8587(17)30015-3 [published Online First: 20170124]

15. Group WCRCW. World Health Organization cardiovascular disease risk charts: revised models to estimate risk in 21 global regions. The Lancet Global Health 2019;7(10):e1332-e45. doi: 10.1016/S2214-109X(19)30318-3 [published Online First: 2019/09/02]

16. Woodward M, Huxley R, Ueshima H, et al. The Asia Pacific cohort studies collaboration: a decade of achievements. Glob Heart 2012;7(4):343-51. doi: 10.1016/j.gheart.2012.10.001 [published Online First: 20121205]

17. Pylypchuk R, Wells S, Kerr A, et al. Cardiovascular disease risk prediction equations in 400 000 primary care patients in New Zealand: a derivation and validation study. Lancet 2018;391(10133):1897-907. doi: 10.1016/s0140-6736(18)30664-0 [published Online First: 20180504]

18. Wang Y, Liu J, Wang W, et al. The lifetime risk for cardiovascular disease in a Chinese population: the Chinese Multi-Provincial Cohort Study. Eur J Prev Cardiol 2015;22(3):380-8. doi: 10.1177/2047487313516563 [published Online First: 20131211]

19. Suebsamran P, Choenchoopon H, Rojanasaksothorn S, et al. Association between Alcohol Consumption and Pre-Diabetes among 383,442 Thai Population Aged 15 Years and Older in Ubon Ratchathani: Analytical Cross-Sectional Study. J Med Assoc Thai 2016;99 Suppl 1:S35-42.

20. Khalili D, Hadaegh F, Soori H, et al. Clinical usefulness of the Framingham cardiovascular risk profile beyond its statistical performance: the Tehran Lipid and Glucose Study. Am J Epidemiol 2012;176(3):177-86. doi: 10.1093/age/kws204 [published Online First: 20120719]

21. Sudlow C, Gallacher J, Allen N, et al. UK biobank: an open access resource for identifying the causes of a wide range of complex diseases of middle and old age. PLoS Med 2015;12(3):e1001779. doi: 10.1371/journal.pmed.1001779 [published Online First: 20150331]

22. Li J, Liu F, Yang X, et al. Validating World Health Organization cardiovascular disease risk charts and optimizing risk assessment in China. The Lancet Regional Health - Western Pacific 2021;8:100096. doi: <https://doi.org/10.1016/j.lanwpc.2021.100096>
