# Supplementary material for: Discrimination and calibration performances of non-laboratory-based and laboratory-based cardiovascular risk predictions: a systematic review
Source: Open Heart. 2025 Feb 10;12(1):e003147. doi: 10.1136/openhrt-2024-003147 (PMC11815431; doi:10.1136/openhrt-2024-003147)
Supplement: online supplemental file 4 [file openhrt-12-1-s004.docx]

Supplementary: Table 2: Cardiovascular Disease Prediction Equations, Populations, Inputs, and Outcomes

| CVD Risk Equations | Study participants | Risk factors included in the models | Predicted outcomes |
| --- | --- | --- | --- |
| INTERHEART laboratory-based ^1^. | Participants were recruited from 252 centers across 52 countries worldwide for a case-control study, with cases and controls recruited between 1999 and 2003 | Age, Apolipoprotein B: A1, smoking status, second-hand smoke, diabetes, high blood pressure, WHR, psychosocial factors (general stress, depression), dietary factor, physical activity | MI |
|  |  |  |  |
|  |  |  |  |
|  |  |  |  |
| INTERHEART non-laboratory-based ^1^ | Same as above | Age, smoking status, second-hand smoke, self-reported diabetes, self-reported high blood pressure, family history of heart attack, WHR, psychosocial factors (general stress, depression), dietary factors, physical activity | Same as above |
|  |  |  |  |
| PARS ^2^. | Irfan cohort (Iranian adults recruited in 2001 and followed for at least ten years) | Age, sex, total cholesterol, systolic blood pressure, diabetes, smoking, family history of CVD, WHR | Fatal (MI or stroke death) and non-fatal CVD (MI and stroke events) |
|  |  |  |  |
| SPARS ^3^ | Same as above | Age, sex, systolic blood pressure, self-reporting history of diabetes, smoking, WHR | Same as above |
|  |  |  |  |
| EPIC-non-clinical ^4^ | EPIC-Potsdam cohort, German part, started in1998 | Age, sex, waist circumference, smoking status, self-reported hypertension, self-reported diabetes, family history of CVD, and consumption of whole grain, red meat, coffee, high-energy soft drinks, and plant oil | Non-fatal (MI, and stroke), and fatal (MI, and stroke) |
| EPIC-Clinical ^4^ | Same as above | The above non-clinical inputs (age, sex, waist circumference, smoking status, self-reported hypertension, self-reported diabetes, family history of CVD, and consumption of whole grain, red meat, coffee, high-energy soft drinks, and plant oil), along with systolic and diastolic blood pressure, total cholesterol, and HDL cholesterol) | Same as above |
| D’Agostino Framingham non-laboratory-based ^5^ | US (1968 to 1987) | Age, sex, smoking, history of diabetes, systolic blood pressure, treatment for hypertension, BMI | MI, angina, coronary insufficiency, CHD, cerebrovascular event (stroke, TIA), CHF, PAD, CVD death |
|  |  |  |  |
|  |  |  |  |
| D’Agostino Framingham laboratory-based 2008 ^5^ | Same as above | Age, sex, smoking, diabetes, systolic blood pressure, treatment for hypertension, total cholesterol, HDL | Same as above |
|  |  |  |  |
| WHO 2019 laboratory-based ^6^. | Derived using 85 prospective cohorts | Age, smoking status, systolic blood pressure, history of diabetes, and total cholesterol | Fatal or non-fatal MI or CHD; fatal or non-fatal stroke |
|  |  |  |  |
| WHO 2019 Non-laboratory-based ^6^. | Same as above | Age, smoking status, systolic blood pressure, BMI | Same as above |
|  |  |  |  |
| Ueda Globo-risk extension laboratory-based ^7^ | Eight prospective cohorts in the USA: Atherosclerosis Risk in Communities, Cardiovascular Health Study, Framingham Heart Study (original cohort), Framingham Heart Study (offspring cohort), Honolulu Heart Program, Multiple Risk Factor Intervention Trial, Puerto Rico Heart Health Program, and Women’s Health Initiative Clinical Trial. | Age, sex, smoking, blood pressure, diabetes, and total cholesterol. | Fatal and non-fatal CVD (IHD, stroke, MI) |
| Ueda Globo-risk extension non-laboratory-based ^7^ | Same as above | Age, sex, smoking, blood pressure, BMI | Same as above |

PARS: Persian Atherosclerotic Cardiovascular Disease Risk Stratification: SPARS: Simplified Non-Laboratory-Based PARS: EPIC: European Prospective Investigation into Cancer and Nutrition: CVD: Cardiovascular Disease: WHR: Waist-Hip Ratio: MI: Myocardial Infarction: CHD: Coronary Heart Disease: TIA: Transient Ischemic Attack: CHF: Congestive Heart Failure: PAD: Peripheral Artery Disease: IHD: Ischemic Heart Disease: BMI: Body Mass Index: HDL: High-Density Lipoprotein

**Reference**

1. McGorrian C, Yusuf S, Islam S, et al. Estimating modifiable coronary heart disease risk in multiple regions of the world: the INTERHEART Modifiable Risk Score. *Eur Heart J* 2011;32(5):581-9. doi: 10.1093/eurheartj/ehq448 [published Online First: 20101222]

2. Sarrafzadegan N, Hassannejad R, Marateb HR, et al. PARS risk charts: A 10-year study of risk assessment for cardiovascular diseases in Eastern Mediterranean Region. *PLoS One* 2017;12(12):e0189389. doi: 10.1371/journal.pone.0189389

3. Hassannejad R, Mansourian M, Marateb H, et al. Developing Non-Laboratory Cardiovascular Risk Assessment Charts and Validating Laboratory and Non-Laboratory-Based Models. *Global heart* 2021;16(1):58-58. doi: 10.5334/gh.890

4. Schiborn C, Kühn T, Mühlenbruch K, et al. A newly developed and externally validated non-clinical score accurately predicts 10-year cardiovascular disease risk in the general adult population. *Sci Rep* 2021;11(1):19609. doi: 10.1038/s41598-021-99103-4 [published Online First: 20211004]

5. D'Agostino RB, Sr., Vasan RS, Pencina MJ, et al. General cardiovascular risk profile for use in primary care: the Framingham Heart Study. *Circulation* 2008;117(6):743-53. doi: 10.1161/circulationaha.107.699579 [published Online First: 20080122]

6. Group WCRCW. World Health Organization cardiovascular disease risk charts: revised models to estimate risk in 21 global regions. *The Lancet Global health* 2019;7(10):e1332-e45. doi: 10.1016/S2214-109X(19)30318-3 [published Online First: 2019/09/02]

7. Ueda P, Woodward M, Lu Y, et al. Laboratory-based and office-based risk scores and charts to predict 10-year risk of cardiovascular disease in 182 countries: a pooled analysis of prospective cohorts and health surveys. *Lancet Diabetes Endocrinol* 2017;5(3):196-213. doi: 10.1016/s2213-8587(17)30015-3 [published Online First: 20170124]
