# Supplementary material for: Discrimination and calibration performances of non-laboratory-based and laboratory-based cardiovascular risk predictions: a systematic review
Source: Open Heart. 2025 Feb 10;12(1):e003147. doi: 10.1136/openhrt-2024-003147 (PMC11815431; doi:10.1136/openhrt-2024-003147)
Supplement: online supplemental file 5 [file openhrt-12-1-s005.docx]

Supplementary: Table 3: Summary of Calibration Model Performance Measures for Externally Validated Laboratory-Based and Non-Laboratory-Based Equations

| Author, Year | Equation | Sex | Calibration results | |
| --- | --- | --- | --- | --- |
|  | Populations for external validation |  |  |  |
|  |  |  | Non-laboratory-based | Laboratory-based |
| Schiborn C et al. 2021 | EPIC-Potsdam |  |  |  |
|  | Germany-Heidelberg | Both | The CP was well-calibrated for the majority of individuals in the lower nine deciles of predicted risk, while it slightly overestimated the risk in the highest decile. | The CP was well-calibrated for the majority of individuals in the lower nine deciles of predicted risk, while it slightly overestimated the risk in the highest decile |
|  |  |  | O: E ratio=1.05, 95%CI (0.97-1.13) | O: E ratio=1.11, 95%CI (1.03-1.20), |
|  | D' Agostino Framingham |  |  |  |
|  | Germany -Heidelberg | Both | CP showed a substantial overestimation | CP showed a substantial overestimation |
|  | Germany- Potsdam | Both | CP showed a substantial overestimation | CP showed a substantial overestimation |
| Albarqouni L et al 2019 | D' Agostino Framingham |  |  |  |
|  | Australia | Female | CP showed an overestimation of the risk | CP showed an overestimation of the risk |
|  |  | Male | CP showed an overestimation of the risk | CP showed an overestimation of the risk |
| Al−Shamsi S et al 2020 | D' Agostino Framingham |  |  |  |
|  | United Arab Emirates | Female | CP showed an overestimation of the risk | CP showed an overestimation of the risk |
|  |  | Male | CP showed an overestimation of the risk | CP showed an overestimation of the risk |
| Kariuki JK et al 2017 | D' Agostino Framingham |  |  |  |
|  | USA | Female | Hosmer–Lemeshow goodness-of-fit = 14.2 (p-value = 0.11); good. | Hosmer–Lemeshow goodness-of-fit = 10.5 (p-value = 0.31); good. |
|  |  | Male | Hosmer–Lemeshow goodness-of-fit = 25.8 (p-value = 0.002); poor.  CP showed an overestimation of the risk in the 2nd decile. | Hosmer–Lemeshow goodness-of-fit = 21.8 (p-value = 0.01); poor.  CP showed an overestimation of the risk in the 1st, 2nd, 3rd, and 4th deciles. |
|  | INTERHEART |  |  |  |
| Joseph P et al 2018 | Africa | Both | CS = 0.75, 95%CI (0.36-1.15) | CS= 0.98, 95%CI  (0.66-1.30) |
|  | China | Both | CS= 0.81, 95%CI (0.71-0.91) | CS= 0.88, 95%CI (0.78-0.98) |
|  | Middle East | Both | CS = 1.06, 95%CI (0.86-1.26) | CS = 1.41, 95%CI (1.18-1.63) |
|  | North America/Europe | Both | CS = 0.77, 95%CI (0.68-0.87) | CS = 1.04 95%CI (0.93-1.15) |
|  | South America | Both | CS = 0.87, 95%CI (0.77-0.98) | CS = 1.11, 95%CI (0.97-1.24) |
|  | South Asia | Both | CS = 0.75, 95%CI (0.65-0.86) | CS = 1.04, 95%CI (0.95-1.13) |
|  | Southeast Asia | Both | CS = 0.92, 95%CI (0.72-1.12) | CS = 0.99, 95%CI (0.76-1.22) |
| Hassannejad R et al. 2021 | PARS/SPARS |  |  |  |
|  | Iran | Both | slightly overestimated the event rate | slightly overestimated the event rate |
|  |  |  | Nam-D'Agostino χ² = 29.89, p-value = 0.001 | Nam-D’Agostino χ2= 28.57, p-value= 0.001 |
| Ueda, P et al. 2017 | Globo-risk extension |  |  |  |
|  | Australia, Iran, Scotland | Both | not reported | not reported |
| WHO CVD RCWG 2019 | WHO 2019 |  |  |  |
|  | Australia, China, Japan, Singapore, New Zealand, Iran, Thailand, and UK | Both | not reported | not reported |
| Li J et al. 2021 | WHO 2019 |  |  |  |
|  | China | Male | Calibration χ² = 388.18, p-value < 0.001 | Calibration χ² = 321.55, p-value < 0.001 |
|  |  | Women | Calibration χ² = 439.99, p-value < 0.001 | Calibration χ² = 280.69, p-value < 0.001 |

RCWG, Research Chart Working Group; CP, calibration plot; CS, calibration slope, O:E, observed: expected.
