# Supplementary material for: Discrimination and calibration performances of non-laboratory-based and laboratory-based cardiovascular risk predictions: a systematic review
Source: Open Heart. 2025 Feb 10;12(1):e003147. doi: 10.1136/openhrt-2024-003147 (PMC11815431; doi:10.1136/openhrt-2024-003147)
Supplement: online supplemental file 6 [file openhrt-12-1-s006.docx]

Supplementary appendix

This appendix is a part of the original manuscript

Supplement to: Yihun Mulugeta Alemu, Sisay Mulugeta Alemu, Nasser Bagheri, Kinley Wangdi, Dan Chateau “Discrimination and calibration performances of non-laboratory-based and laboratory-based cardiovascular risk predictions: a systematic review”

**Discrimination and calibration performances of non-laboratory-based and laboratory-based cardiovascular risk predictions: a systematic review**

Yihun Mulugeta Alemu, Sisay Mulugeta Alemu, Nasser Bagheri, Kinley Wangdi, Dan Chateau

Supplementary appendices

[Appendix A: Details of the search strategies 3](#_Toc166233576)

[Appendix B: PRISMA Checklist 6](#_Toc166233577)

[PRISMA checklist for abstract 6](#_Toc166233578)

[PRISMA checklist for the main body 6](#_Toc166233579)

[Appendix C: PRISMA Flowchart 10](#_Toc166233580)

[Appendix: D Risk of Bias Assessment Tool 11](#_Toc166233581)

[Appendix: E Studies excluded for reasons 12](#_Toc166233582)

# Appendix A: Details of the search strategies

| **Systematic review search strategies** |
| --- |
| **PubMed**  #1 "Models, Cardiovascular"[Mesh] =35,026  #2 “laboratory-based” OR “laboratory based” [tw] OR “non-laboratory-based” OR “nonlaboratory based” OR “risk score*” [tw] OR “cardiovascular risk score*”[tw] OR “cardiovascular risk equation*”[tw] OR “cardiovascular risk prediction*”[tw] OR “non-fatal stroke”[tw] OR “fatal stroke”[tw] OR “myocardial infarction”[tw] OR “nonfatal myocardial infarction”[tw] OR “nonfatal myocardial infarction”[tw] OR “ischemic heart disease”[tw] OR “cardiovascular death*”[tw] OR “congestive heart failure”[tw] OR “coronary bypass*”[tw] OR “percutaneous angioplasty*”[tw] OR “angina*”[tw] OR “coronary insufficiency*”[tw] OR “coronary heart disease death *”[tw] OR “transient ischemic attack*”[tw] OR “peripheral vascular disease*”[tw] OR “ischemic heart disease*”[tw] = 507,347  #3 #1 OR #2=547,129  #4 "Risk Factors"[Mesh] OR "Body Mass Index"[Mesh] OR "Cholesterol"[Mesh]= 1,219,627  #5 “10 year risk*”[tw] OR “5 year risk*”[tw] OR "comparison"[tw] = 1,586,134  #6 #4 AND #5= 54,030  #7 #3 AND #6=6,014 |
| **WEB of SCIENCE**  #1 "laboratory-based" OR " cardiovascular risk score*" OR "non-laboratory-based*" OR "cardiovascular risk equation*" OR "cardiovascular risk prediction*" OR "coronary heart diseases event*" OR "cardiovascular risk estimation" OR "body mass index cardiovascular" OR "cardiac risk factors " OR "coronary disease risk" OR "non-fatal stroke" OR "nonfatal myocardial infarction" OR "hypertensive ischemic heart disease" OR "Harvard NHANES equation" OR " Framingham 2008 risk score" OR " Framingham 1991 CVD score" OR " SCORE high-risk score" OR " SCORE low-risk score" OR " CUORE risk score" OR "pooled cohort equation " OR "pooled cohort equation " OR " Framingham non-laboratory-based algorithm" OR "office based cardiovascular score" OR " WHO/ISH cardiovascular score" OR " Globorisk score" OR " Swedish consultation-based method" OR " UK General Practice model" OR " UK GP model" (Topic) = 21,880  #2 "correlation" OR "comparison" OR "Spearman*" OR " Pearson" OR "association" OR "estimation" OR "agreement" OR "concordance" OR "kappa" OR "c index" OR "c statistics" OR "discrimination" OR "calibration" OR "external validation" OR "forecast" "probability" OR "mathematical model" (Topic)= 8,634,045  #3 #1 AND #2 = 7,581  #4 “risk score*” OR “risk equation*” OR “risk prediction” OR “risk model” (Topic)= 77,918  #5 "cardiovascular diseases*" OR " laboratory-based*" OR "non-laboratory-based *" OR "nonlaboratory based*" OR "cardiovascular risk" OR " clinical laboratory techniques " OR " cardiovascular model" OR "cholesterol" OR " lipid-based" OR " lipid-based" OR " blood-based" OR " non-blood based" (Topic)= 523,112  #6 "10-year risk*" OR "10-year risk*" OR "5-year risk*" OR "5-year risk*" OR "ten-year risk*" OR "five-year risk*" OR "stroke" OR "fatal stroke" OR "nonfatal stroke" OR "myocardial infarction" OR "fatal myocardial infarction" OR "non-fatal myocardial infarction" OR " cardiovascular death" OR "congestive heart failure" OR " coronary bypass " OR "angina" OR "percutaneous angioplasty" OR " coronary insufficiency " OR " coronary insufficiency " OR "coronary heart disease death" OR " transient ischemic attack" OR "transient ischemic attack*" OR "peripheral vascular disease" OR "hypertensive ischemic heart disease" (Topic) = 819,998  #7 #4 AND #5 AND #6= 3,495  #8 #3 OR #7= 10,572 |
| **Scopus**  #1 TITLE-ABS-KEY (cardiovascular OR " risk prediction" OR "risk score" OR laboratory OR “laboratory-based” OR “non-laboratory-based” OR “non-blood-based” OR “blood-based” OR “lipid-based” OR “non-lipid-based” OR “fatal CVD event” OR “non-fatal CVD event” OR “non-fatal CHD” OR “PVD” OR “IHD” OR “TIA” OR “PTCA” OR “CHF” OR “CVD death” OR “MI”)= 3,361,581  #2 TITLE-ABS-KEY ( "Harvard NHANES equation" OR " Framingham 2008 risk score" OR " Framingham 1991 CVD score" OR " SCORE high-risk score" OR " SCORE low-risk score" OR " CUORE risk score" OR "pooled cohort equation " OR "pooled cohort equation " OR " Framingham non-laboratory-based algorithm" OR "office based cardiovascular score" OR " WHO/ISH cardiovascular score" OR " Globorisk score" OR "Swedish consultation-based method" OR "UK General Practice model" OR "UK GP model" )= 552  # 3 #1 AND #2=543 |
| **Google Scholar**  #1 "laboratory-based" OR " cardiovascular risk score*" OR "non-laboratory-based*" OR "cardiovascular risk equation*" OR "cardiovascular risk prediction*" OR "coronary heart diseases event*" OR "cardiovascular risk estimation" OR "body mass index cardiovascular" OR "cardiac risk factors " OR "coronary disease risk" OR "non-fatal stroke" OR "nonfatal myocardial infarction" OR "hypertensive ischemic heart disease" OR "Harvard NHANES equation" OR "Framingham 2008 risk score" OR " Framingham 1991 CVD score" OR " SCORE high-risk score" OR " SCORE low-risk score" OR " CUORE risk score" OR "pooled cohort equation" OR "pooled cohort equation " OR "Framingham non-laboratory-based algorithm" OR "office based cardiovascular score" OR " WHO/ISH cardiovascular score" OR " Globorisk score" OR " Swedish consultation-based method" OR " UK General Practice model" OR " UK GP mode” **=** 56 |
| **ProQuest Dissertations & Theses Global**  #1 ("laboratory-based risk score*" OR "non-laboratory-based risk score*" OR "cardiovascular risk equation*" OR "cardiovascular risk prediction*") AND ("comparison") AND ("Agreement”) =348 |

# Appendix B: PRISMA Checklist

| **Section and Topic** | **Item #** | **Checklist item** | **Reported (Yes/No)** |
| --- | --- | --- | --- |
| **TITLE** | | |  |
| Title | 1 | Identify the report as a systematic review. | Yes |
| **BACKGROUND** | | |  |
| Objectives | 2 | Provide an explicit statement of the review's main objective(s) or question(s). | Yes |
| **METHODS** | | |  |
| Eligibility criteria | 3 | Specify the inclusion and exclusion criteria for the review. | Yes |
| Information sources | 4 | Specify the information sources (e.g., databases, registers) used to identify studies and when each was last searched. | Yes |
| Risk of bias | 5 | Specify the methods used to assess the risk of bias in the included studies. | Yes |
| Synthesis of results | 6 | Specify the methods used to present and synthesize results. | Yes |
| **RESULTS** | | |  |
| Included studies | 7 | Give the total number of included studies and participants and summarise relevant characteristics of studies. | Yes |
| Synthesis of results | 8 | Present results for main outcomes, preferably indicating the number of included studies and participants. Report the summary estimate and confidence/credible interval if a meta-analysis was done. If comparing groups, indicate the direction of the effect (i.e. which group is favored). | Yes |
| **DISCUSSION** | | |  |
| Limitations of evidence | 9 | Provide a summary of the limitations of the evidence included in the review (e.g. study risk of bias, inconsistency, and imprecision). | Yes |
| Interpretation | 10 | Provide a general interpretation of the results and important implications. | Yes |
| **OTHER** | | |  |
| Funding | 11 | Specify the primary source of funding for the review. | Yes |
| Registration | 12 | Provide the register name and registration number. | Yes |

## PRISMA checklist for abstract

## PRISMA checklist for the main body

| **Section and Topic** | **Item #** | **Checklist item** | | **The location where the item is reported** |
| --- | --- | --- | --- | --- |
| **TITLE** | | | |  |
| Title | 1 | Identify the report as a systematic review. | | pp 1 |
| **ABSTRACT** | | | |  |
| Abstract | 2 | See the PRISMA 2020 for Abstracts checklist. | | Completed |
| **INTRODUCTION** | | | |  |
| Rationale | 3 | Describe the rationale for the review in the context of existing knowledge. | | pp 3 |
| Objectives | 4 | Provide an explicit statement of the objective(s) or question(s) the review addresses. | | pp 3 |
| **METHODS** | | | |  |
| Eligibility criteria | 5 | Specify the inclusion and exclusion criteria for the review and how studies were grouped for the syntheses. | | pp 4 |
| Information sources | 6 | Specify all databases, registers, websites, organizations, reference lists, and other sources searched or consulted to identify studies. Specify the date when each source was last searched or consulted. | | pp 4 |
| Search strategy | 7 | Present the full search strategies for all databases, registers, and websites, including any filters and limits used. | | Appendix A |
| Selection process | 8 | Specify the methods used to decide whether a study met the review's inclusion criteria, including how many reviewers screened each record and each report retrieved, whether they worked independently, and if applicable, details of automation tools used in the process. | | pp 4,5 |
| Data collection process | 9 | Specify the methods used to collect data from reports, including how many reviewers collected data from each report, whether they worked independently, any processes for obtaining or confirming data from study investigators, and if applicable, details of automation tools used in the process. | | pp 4,5 |
| Data items | 10a | List and define all outcomes for which data were sought. Specify whether all results that were compatible with each outcome domain in each study were sought (e.g. for all measures, time points, analyses), and if not, the methods used to decide which results to collect. | | pp 5 |
|  | 10b | List and define all other variables for which data were sought (e.g. participant and intervention characteristics, funding sources). Describe any assumptions made about any missing or unclear information. | | pp 5 |
| Study risk of bias assessment | 11 | Specify the methods used to assess the risk of bias in the included studies, including details of the tool(s) used, how many reviewers assessed each study whether they worked independently, and if applicable, details of automation tools used in the process. | | pp 4,5 |
| Effect measures | 12 | Specify for each outcome the effect measure(s) (e.g. risk ratio, mean difference) used in the synthesis or presentation of results. | | pp 5,6 |
| Synthesis methods | 13a | Describe the processes used to decide which studies were eligible for each synthesis (e.g. tabulating the study intervention characteristics and comparing against the planned groups for each synthesis (item #5)). | | pp 5,6 |
|  | 13b | Describe any methods required to prepare the data for presentation or synthesis, such as handling of missing summary statistics, or data conversions. | | pp 5,6 |
|  | 13c | Describe any methods used to tabulate or visually display the results of individual studies and syntheses. | | pp 5,6 |
|  | 13d | Describe any methods used to synthesize results and provide a rationale for the choice(s). If meta-analysis was performed, describe the model(s), method(s) to identify the presence and extent of statistical heterogeneity, and software package(s) used. | | pp 4,5 |
|  | 13e | Describe any methods used to explore possible causes of heterogeneity among study results (e.g., subgroup analysis, meta-regression). | | NA |
|  | 13f | Describe any sensitivity analyses conducted to assess the robustness of the synthesized results. | | NA |
| Reporting bias assessment | 14 | Describe any methods used to assess the risk of bias due to missing results in a synthesis (arising from reporting biases). | | NA |
| Certainty assessment | 15 | | Describe any methods used to assess certainty (or confidence) in the body of evidence for an outcome. | NA |
| **RESULTS** | | | |  |
| Study selection | 16a | | Describe the results of the search and selection process, from the number of records identified in the search to the number of studies included in the review, ideally using a flow diagram. | Appendix C |
|  | 16b | | Cite studies that might appear to meet the inclusion criteria, but which were excluded, and explain why they were excluded. | Appendix E |
| Study characteristics | 17 | | Cite each included study and present its characteristics. | Table 1, pp 6,7 |
| Risk of bias in studies | 18 | | Present assessments of risk of bias for each included study. | Appendix D |
| Results of individual studies | 19 | | For all outcomes, present, for each study: (a) summary statistics for each group (where appropriate) and (b) an effect estimate and its precision (e.g., confidence/credible interval), ideally using structured tables or plots. | Figure 1,2, and Table 2, 3 |
| Results of syntheses | 20a | | For each synthesis, briefly summarise the characteristics and risk of bias among contributing studies. | Table 1 |
|  | 20b | | Present results of all statistical syntheses conducted. If meta-analysis was done, present for each the summary estimate and its precision (e.g. confidence/credible interval) and measures of statistical heterogeneity. If comparing groups, describe the direction of the effect. | Figure 1, 2, Table 2, 3 |
|  | 20c | | Present results of all investigations of possible causes of heterogeneity among study results. | NA |
|  | 20d | | Present results of all sensitivity analyses conducted to assess the robustness of the synthesized results. | NA |
| Reporting biases | 21 | | Present assessments of risk of bias due to missing results (arising from reporting biases) for each synthesis assessed. | NA |
| Certainty of evidence | 22 | | Present assessments of certainty (or confidence) in the body of evidence for each outcome assessed. | Figure 1,2 |
| **DISCUSSION** | | | |  |
| Discussion | 23a | | Provide a general interpretation of the results in the context of other evidence. | pp 8, 9 |
|  | 23b | | Discuss any limitations of the evidence included in the review. | pp 9 |
|  | 23c | | Discuss any limitations of the review processes used. | pp 9 |
|  | 23d | | Discuss the implications of the results for practice, policy, and future research. | pp 9 |
| **OTHER INFORMATION** | | | |  |
| Registration and protocol | 24a | | Provide registration information for the review, including the register name and registration number, or state that the review was not registered. | pp 4 |
|  | 24b | | Indicate where the review protocol can be accessed, or state that a protocol was not prepared. | pp 4 |
|  | 24c | | Describe and explain any amendments to information provided at registration or in the protocol. | NA |
| Support | 25 | | Describe sources of financial or non-financial support for the review, and the role of the funders or sponsors in the review. | pp 10 |
| Competing interests | 26 | | Declare any competing interests of review authors. | pp 10 |
| Availability of data, code, and other materials | 27 | | Report which of the following are publicly available and where they can be found template data collection forms; data extracted from included studies; data used for all analyses; analytic code; and any other materials used in the review. | pp 10 |

# Appendix C: PRISMA Flowchart

**IDENTIFICATION**

Record identified through database searching (n= 17,533)

PubMed: 6,014 Google scholar:56

WEB of SCIENCE: 10,572 Scopus: 543

ProQuest Dissertations & Theses Global: 348

Duplicates removed =1,529

**SCREENING**

Records screened (16,004)

Records excluded on basis of abstract (n= 15,770)

Full text excluded (n=226)

- risk estimates only (n=19)

- qualitative evaluation of equation (n=1)

- laboratory-based only (n=102)

- duplicates sample (n=1)

- predictor focused other than non-laboratory (n=27)

- no specific measures/ranges (n=1)

- without external validation(n=7)

- interventions evaluation following the equation (n=11)

- non-laboratory-based only (n=5)

- event at the baseline (n=11)

- not measured model performances (n=14)

- validated after recalibration (n=2)

- reporting correlation/kappa(n=25)

**ELIGIBILITY**

Articles assessed for eligibility (n=234)

**INCLUSION**

Additional articles identified from references of published studies (n=1)

Articles included in

the analysis (n=9)

# Appendix: D Risk of Bias Assessment Tool

Predictive model risk of bias assessment tools (PROBAST) is used for risk of bias assessment.

List of domains and signaling questions used for PROBAST.

| **Domain** | **Signaling question** |
| --- | --- |
| Participant selection | 1. Were appropriate data sources used, e.g. cohort, RCT, or nested case-control study data? |
|  | 2. Were all inclusions and exclusions based on characteristics of participants appropriate (e.g. comorbidities, treatment)? |
| Predictors | 1. Were predictors defined and assessed in a similar way for all participants? |
|  | 2. Were predictor assessments made without knowledge of outcome data? |
|  | 3. Are all predictors available at the time the model is used? |
|  | 4. Were predictors defined and assessed in the same way as in the original Framingham model? |
| Outcome | 1. Was a pre-specified outcome definition used? |
|  | 2. Were predictors excluded from the outcome definition? |
|  | 3. Was the outcome defined and determined in a similar way for all participants? |
|  | 4. Was the outcome determined without knowledge of predictor information? |
|  | 5. Are you confident that the outcome has been correctly measured for all patients (e.g. no outcomes are missed)? |
| Sample size and participant flow | 1. Were there a reasonable number of outcome events? |
|  | 2. Was the time interval between predictor assessment and outcome determination appropriate? |
|  | 3. Were all enrolled participants included in the analysis? |
|  | 4. Were participants with missing data handled appropriately? |
| Analysis | 1. Were any complexities in the data (e.g. censoring, competing risks) accounted for appropriately? |
|  | 2. Was the model *not* recalibrated before validation? |

# Appendix: E Studies excluded for reasons

| 1. Sr.no. | Title | Reasons |
| --- | --- | --- |
|  | Comparability of total cardiovascular disease risk estimates using laboratory and non-laboratory-based assessments in urban-dwelling South Africans: The CRIBSA study | duplicate sample |
|  | Evaluation of the Framingham risk score and pooled cohort risk equation for prediction of cardiovascular risk in low resource areas: Insights from Asian rural population | no full document is available |
|  | 10-Year Cardiovascular Disease Risk Estimation Based on Lipid Profile-Based and BMI-Based Framingham Risk Scores across Multiple Sociodemographic Characteristics: The Malaysian Cohort Project | compare risk estimates only |
|  | Anthropometric measurements of general and central obesity and the prediction of cardiovascular disease risk in women: a cross-sectional study | predictors focused |
|  | Correlation between the Framingham risk score and intima-media thickness: The Paroi Art´erielle et Risque Cardio-vascular (PARC) Study | carotid intima thickness |
|  | Is lipid accumulation product a better cardiovascular risk predictor in elderly individuals than anthropometric measures? | anthropometric predictors |
|  | Risk assessment in the prevention of cardiovascular disease in low-resource settings | review |
|  | Comparative risk assessment for the development of cardiovascular diseases in the Hungarian general and Roma population | only laboratory-based |
|  | Comparison of lab-and non-lab-based absolute cardiovascular disease risk scores in rural India | no full text is available |
|  | Absolute cardiovascular risk scores and medication use in rural India: a cross-sectional study | compare laboratory-based only |
|  | Factors influencing the implementation of cardiovascular risk scoring in primary care: a mixed-method systematic review | review |
|  | Primary prevention of cardiovascular disease using validated risk scores: A systematic review | review |
|  | Circulating Biomarkers for Predicting Cardiovascular Disease Risk; a Systematic Review and Comprehensive Overview of Meta-Analyses | review |
|  | Global cardiovascular risk assessment in the primary prevention of cardiovascular disease in adults: systematic review of systematic reviews | review and review |
|  | Cardiovascular risk factors, cardiovascular disease, and COVID-19: an umbrella review of systematic reviews | review |
|  | Circulating Apolipoprotein E Concentration and Cardiovascular Disease Risk: Meta-analysis  of Results from Three Studies | review |
|  | A community-based cross-sectional study on the prevalence of dyslipidemias and 10 years cardiovascular risk scores in adults in Asmara, Eritrea | lipid predictor only |
|  | Risk-factor profiles for chronic diseases of lifestyle and metabolic syndrome in an urban and rural setting in South Africa | risk profile only |
|  | Cardiovascular risk assessment tools in Asia | equation evaluations (qualitative) |
|  | Comparisons of the Framingham and Pooled Cohort Equation Risk Scores for Detecting Subclinical Vascular Disease in Blacks Versus Whites | compare laboratory-based only |
|  | Comparative performance of cardiovascular risk prediction models in people living with HIV | compare laboratory-based only |
|  | Cardiovascular risk prediction in HIV-infected patients: comparing the Framingham, atherosclerotic cardiovascular disease risk score (ASCVD), Systematic Coronary Risk  Evaluation for the Netherlands (SCORE-NL) and Data Collection on Adverse Events of Anti-HIV Drugs (D: A:D) risk prediction models | compare laboratory-based only |
|  | Comparison of four international cardiovascular disease prediction models and the prevalence of eligibility for lipid-lowering therapy in HIV-infected patients on antiretroviral therapy | compare laboratory-based only |
|  | Comparison of ACC/AHA and ESC Guideline Recommendations Following Trial Evidence for Statin Use in Primary Prevention of Cardiovascular Disease: Results from the Population-Based Rotterdam Study | compare for initiation of intervention |
|  | Ten-year cardiovascular risk among Bangladeshi population using non-laboratory-based risk chart of the World Health Organization: Findings from a nationally representative survey | non-laboratory-based only |
|  | Estimation of total cardiovascular risk using the 2019 WHO CVD prediction charts and comparison of population-level costs based on alternative drug therapy guidelines | non-laboratory-based only |
|  | Estimated total cardiovascular risk in a rural area of Bangladesh: a household level cross-sectional survey done by local community health workers | non-laboratory-based only |
|  | Estimation of 10-Year Risk of Cardiovascular Diseases Using WHO Risk Prediction Charts: A Population-Based Study in Southern Iran | laboratory-based only |
|  | Evaluation of cardiovascular diseases risk calculators for CVD prevention and management: scoping review | review |
|  | Comparison of different cardiovascular risk score calculators for cardiovascular risk prediction and guideline-recommended statin uses | laboratory-based only |
|  | Comparison of Application of the ACC/AHA Guidelines, Adult Treatment Panel III Guidelines, and European Society of Cardiology Guidelines for Cardiovascular Disease Prevention in a European Cohort | laboratory-based only |
|  | Recalibration of the Framingham risk score for predicting 10-year risk of cardiovascular events: A non-concurrent rural cohort study from Tamil Nadu | compare risk estimates only |
|  | Comparison of Framingham Cardiovascular Risk Criteria and ASCVD Score in Iranian Obese Patients | laboratory-based only |
|  | Comparison of Framingham Risk Scores (FRS), Joint British Society (JBS3), and American College of Cardiology/American Heart Association (ACC/AHA) Cardiovascular Risk Scores Among Adults With First Myocardial Infarction | laboratory-based only |
|  | A cross-sectional validation study comparing the accuracy of different risk scores in assessing the risk of acute coronary syndrome among patients in a tertiary care hospital in Kerala | compare risk estimates |
|  | Assessment of total cardiovascular risk using WHO/ISH risk prediction charts in three low- and middle-income countries in Asia | compare risk estimates |
|  | Agreement between the SCORE and D’Agostino Scales for the Classification of High Cardiovascular Risk in Sedentary Spanish Patients | compare laboratory-based only |
|  | Sedentary lifestyle and Framingham risk scores: a population-based study in Riyadh city, Saudi Arabia | CVD risk estimate and predictor-focused |
|  | Comparison of Cardiac Risk Scores among the East Mediterranean and South Asian Population | laboratory-based only |
|  | Cardiovascular Disease Risk Factors and 10-Year Risk of Cardiovascular Events among Women over the Age of 40 Years in an Urban Underprivileged Area of Bangalore City | risk estimate and predictors focused |
|  | Agreement between 2017 ACC/AHA Hypertension Clinical Practice Guidelines and Seventh Report of the Joint National Committee Guidelines to Estimate Prevalence of Postmenopausal Hypertension in a Rural Area of Bangladesh: A Cross-Sectional Study | One risk factor /hypertension/ focused |
|  | Variation among cardiovascular risk calculators in relative risk increases with identical risk factor increases | hypothetical data and compare laboratory-based only |
|  | A Comparison of Statin Treatment Algorithms Based on the ACC/AHA and Philippine Guidelines for Primary Prevention of Dyslipidemia in Statin-Naive Filipino Patients | intervention based comparison |
|  | Prediction of cardiovascular disease risk among low-income urban dwellers in metropolitan Kuala Lumpur, Malaysia | risk estimates and predictors |
|  | Body Composition Indices and Predicted Cardiovascular Disease Risk Profile among Urban Dwellers in Malaysia | risk estimates and predictors |
|  | Total cardiovascular risk for the next 10 years among the rural population of Nepal using the WHO/ISH risk prediction chart | risk estimates and predictors |
|  | Comparison of three different methods of assessing cardiovascular disease risk in New Zealanders with Type 2 diabetes mellitus | compare laboratory-based only |
|  | Comparing six cardiovascular Risk prediction models in Haiti: implications for identifying high-risk individuals for Primary prevention | range of correlation reported, but not the exact correlation values |
|  | Prevalence of traditional cardiovascular risk factors and evaluation of cardiovascular risk using three risk equations in Nigerians living with human immunodeficiency virus | compare risk estimate |
|  | World Health Organization (WHO) and International Society of Hypertension (ISH) risk prediction charts: assessment of cardiovascular risk for prevention and control of cardiovascular disease in low and middle-income countries | equation development and use of equations only, not for comparison |
|  | Ten-year atherosclerosis cardiovascular disease (ASCVD) risk score and its components among an Iranian population | risk estimates only |
|  | Body Weight, Cardiovascular Risk Factors, and Coronary Mortality | predictor focused |
|  | A comparison of cardiovascular risk scores in native and migrant South Asian populations | laboratory-based only |
|  | Assessing 10-year coronary heart disease risk in people with Type 2 diabetes mellitus: Framingham versus United Kingdom Prospective Diabetes Study | laboratory-based only |
|  | Comparison of abdominal obesity measures in predicting 10-year cardiovascular risk in an Iranian adult population using ACC/AHA risk model: A population-based cross-sectional study | compare predictors |
|  | Agreement Among Cardiovascular Disease Risk Calculators | review of laboratory equations |
|  | The Ten-Year Risk Prediction for Cardiovascular Disease for Malaysian Adults Using the Laboratory-Based and Office-Based (Global Risk) Prediction Model | compare risk estimates only |
|  | Cost-effectiveness of the non-laboratory-based Framingham algorithm in primary prevention of cardiovascular disease: A simulated analysis of a cohort of African American adults | compare sensitivity and specificity |
|  | Ambiguity about Selection of Cardiovascular Risk Stratification Tools: Evidence from a North Indian Rural Population | non-laboratory based only |
|  | Estimation of the 10-Year Risk of Cardiovascular Diseases: Using the SCORE, WHO/ISH, and Framingham Models in the Shahrekord Cohort Study in Southwestern Iran | compare laboratory-based only |
|  | Estimation of the Cardiovascular Risk Using World Health Organization/International Society of Hypertension (WHO/ISH) Risk Prediction Charts in a Rural Population of South India | compare risk estimates only |
|  | Risk estimates of cardiovascular diseases in a Sri Lankan community | compare risk estimates only |
|  | Agreement between Framingham, IraPEN, and non-laboratory WHO-EMR risk score calculators for cardiovascular risk prediction in a large Iranian population | compare non-laboratory-based equations |
|  | Differences in the Cardiovascular Risk Assessment in Cardiology Outpatients in Mali: Comparison between Framingham Body Mass Index-Based Tool and Low-Information World Health Organization Chart | compare across equations |
|  | Prediction of Cardiovascular Disease Mortality in a Middle Eastern Country: Performance of the Globo Risk and Score Functions in Four Population-Based Cohort Studies of Iran | compare laboratory-based equations |
|  | The predicted 10-year risk of cardiovascular disease is influenced by the risk equation adopted: | compare risk estimates only |
|  | An assessment of community health workers’ ability to screen for cardiovascular disease risk with a simple, non-invasive risk assessment instrument in Bangladesh, Guatemala, Mexico, and South Africa: an observational study | compare the effectiveness of non-laboratory-based equation |
|  | Cardiovascular risk assessment of South Asians in a religious setting: a feasibility study | compare across equations |
|  | Feasibility of community-based screening for cardiovascular disease risk in an ethnic community: the South Asian Cardiovascular Health Assessment and Management Program (SA-CHAMP) | compare for targeted intervention |
|  | Association of systolic blood pressure levels with cardiovascular events and all-cause mortality among older adults taking antihypertensive medication | predictor comparison |
|  | Cardiometabolic risk factors and Framingham Risk Score in severely obese patients: Baseline data from DieTBra trial | predictor comparison |
|  | Comparisons of the Framingham and ASCVD risk scores for coronary heart disease risk prediction in Chinese men | compare across equation type |
|  | Agreement between cardiovascular disease risk assessment tools: An application to the United Arab Emirates population | compare laboratory-based only |
|  | Cardiometabolic risk in a population of older adults with multiple co-morbidities in  Rural South Africa: the HAALSI (Health and Aging in Africa: longitudinal studies of  INDEPTH communities) study | predictors only |
|  | Place of cardiovascular risk prediction models in South Asians; agreement between Framingham risk score and WHO/ISH risk charts | Only kappa reported, no standard error, no poi, no PCI, no 2x2 or 3x3 risk category measures |
|  | A high correlation between Framingham equations with BMI and with lipids to estimate cardiovascular risk score at baseline in HIV-infected adults in the Temprano trial, ANRS 12136 in CoÃte d'Ivoire | no sufficient information no standard error, no POI, no PCI, no 2x2 or 3x3 category measures |
|  | Cardiovascular risk assessment in type 2 diabetes mellitus: comparison of the World Health Organization/International Society of Hypertension risk prediction charts versus UK Prospective Diabetes Study risk engine | laboratory-based comparison only |
|  | WHO/International Society of Hypertension Risk Prediction charts versus the UK Prospective Diabetes Study risk engine for cardiovascular risk assessment among patients with type 2 diabetes: a comparative study | editorial notes |
|  | Cardiovascular Risk Assessment in Diabetes Mellitus: Comparison of the  General Framingham Risk Profile Versus the World Health Organization/ International Society of Hypertension Risk Prediction Charts in Arabs—Clinical Implications | compare laboratory-based only |
|  | Cardiovascular disease risk profile and management among people 40 years of age and above in Bo, Sierra Leone: A cross-sectional study | focused on predictors for laboratory-based |
|  | Concordance between Two Versions of the World Health Organization/International Society of Hypertension Risk Prediction Chart and Framingham Risk Score among Postmenopausal Women in a Rural Area of Bangladesh | merge risk categories into negative-risk (low risk) and positive-risk (moderate, high, very high) |
|  | Prediction of 10-year atherosclerotic cardiovascular disease risk among community residents in Shanghai, China – a comparative analysis of risk algorithms | laboratory-based only |
|  | Assessment of Short-Term Cardiovascular Risk Among 40 Years and Above Population in a Selected Community of Kathmandu, Nepal | risk estimates only |
|  | The 10-year Absolute Risk of Cardiovascular (CV) Events in Northern Iran: a Population-Based Study | laboratory-based only |
|  | Comparison of cardiovascular risk assessment tools and their guidelines in evaluation of 10-year CVD risk and preventive recommendations: A population-based study | laboratory-based only |
|  | Cardiovascular disease risk prediction by the American College of Cardiology (ACC)/American Heart Association (AHA) Atherosclerotic Cardiovascular Disease (ASCVD) risk score among HIV-infected patients in sub-Saharan Africa | laboratory-based only |
|  | Coronary disease risk assessment in men: Comparison between ASCVD Risk versus Framingham | laboratory-based only |
|  | Comparison of Predicted Cardiovascular Risk Profiles by Different CVD Risk-Scoring Algorithms between HIV-1-Infected and Uninfected Adults | compare risk estimates between the population |
|  | A Comparison of Four Cardiovascular Risk Assessment Instruments in Saudi Patients | compare only risk estimates |
|  | Comparison of cardiovascular risk assessment tools and their guidelines in evaluation of 10-year CVD risk and preventive recommendations: a population study | laboratory-based only |
|  | Guideline-Based Statin Eligibility, Coronary Artery Calcification, and Cardiovascular Events | intervention-based comparison |
|  | Level of agreement between frequently used cardiovascular risk calculators in people living with HIV | compare laboratory-based only |
|  | High Concordance between D:A: Dr and the Framingham Risk Score in Brazilians Living with HIV | laboratory-based only |
|  | Comparison of Three Cardiovascular Risk Scores among HIV-Infected Patients in Korea: The Korea HIV/AIDS Cohort Study | laboratory-based only |
|  | Implications of Cardiovascular Disease Risk Assessment Using the WHO/ISH Risk Prediction Charts in Rural India | compare misclassification, does not compare agreements |
|  | The "Five Risks Algorithm": an easy tool for cardiovascular risk estimation | compare based on laboratory-test |
|  | Use of risk assessment tools to guide decision-making in the primary prevention of atherosclerotic cardiovascular disease circulation | laboratory-based only |
|  | Performance of the Framingham risk models and pooled cohort equations for predicting 10-year risk of cardiovascular disease: a systematic review and meta-analysis | review |
|  | Prediction models for cardiovascular disease risk in the general population: systematic review | review |
|  | Comparisons of established risk prediction models for cardiovascular disease: systematic review | review |
|  | Predicting the 10-Year Risks of Atherosclerotic Cardiovascular Disease in Chinese Population | compare laboratory-based only |
|  | Comparison of the Framingham Risk Score, SCORE, and WHO/ISH cardiovascular risk prediction models in an Asian population | compare laboratory-based only |
|  | Prediction of Cardiovascular Disease Mortality in a Middle Eastern Country: Performance of the Globorisk and Score Functions in Four Population-Based Cohort Studies of Iran | compare laboratory-based only |
|  | Validation of the Framingham general cardiovascular risk score in a multiethnic Asian population: a retrospective cohort study | compare laboratory-based only |
|  | Development and Validation of Improved Algorithms for the Assessment of Global Cardiovascular Risk in Women | require laboratory factors |
|  | Performance of atherosclerotic cardiovascular risk prediction models in a  rural Northern Chinese population: Results from the Fangshan Cohort Study | compare laboratory-based only |
|  | Cardiovascular risk prediction tools for populations in Asia | low information, but require a total cholesterol test |
|  | WHO cardiovascular disease risk prediction model performance in 10 regions, China | validate non-laboratory based only |
|  | Cardiovascular disease risk prediction models in the Chinese population‑ a systematic review and meta‑analysis | review |
|  | Performance of the SCORE and Globo risk cardiovascular risk prediction models | compare non-laboratory only |
|  | The additive EuroSCORE | review |
|  | Review and evaluation of performance measures for survival prediction models in external validation settings | review |
|  | Clinical Usefulness of the Framingham Cardiovascular Risk Profile Beyond Its Statistical Performance | laboratory-based only |
|  | Evaluation of the Performance of Survival Analysis Models: Discrimination and Calibration Measures | method |
|  | Derivation, internal validation, and recalibration of a cardiovascular risk score for Latin America and the Caribbean (Globorisk-LAC): A pooled analysis of cohort studies | internal validation only |
|  | An office-based cardiovascular prediction model developed and validated in cohort studies of a middle-income country | cross-validation only and sample duplicate |
|  | External validation of two Framingham cardiovascular risk equations and the Pooled Cohort equations: A nationwide registry analysis | compare laboratory-based only |
|  | Equalization of four cardiovascular risk algorithms after systematic recalibration: an individual-participant meta-analysis of 86 prospective studies | compare laboratory-based only |
|  | Evaluation of the performance of existing non-laboratory Cardiovascular risk assessment algorithms | qualitative evaluation |
|  | Nontraditional Risk Factors in Cardiovascular Disease Risk Assessment | scopes review |
|  | Validation of the general Framingham Risk Score (FRS), SCORE2, revised PCE, and WHO CVD risk scores in an Asian population | compare laboratory-based only |
|  | Prediction for cardiovascular diseases based on laboratory data: An analysis of random forest model | mix laboratory-based and non-laboratory predictor |
|  | Anthropometric measures in cardiovascular disease prediction: comparison of laboratory-based versus non-laboratory-based model | no external validation |
|  | Polygenic risk scores in cardiovascular risk prediction: A cohort study and modeling analyses | laboratory predictor only |
|  | Cardiovascular Risk and Events in 17 Low-, Middle-, and High-Income Countries | risk estimates only |
|  | Development and validation of a cardiovascular disease risk-prediction model using population health surveys: the Cardiovascular Disease Population Risk Tool (CVDPoRT) | no separate non-laboratory-based equation |
|  | Assessing risk of myocardial infarction and stroke: new data from the Prospective Cardiovascular Münster (PROCAM) study | laboratory-based only |
|  | Laboratory and non-laboratory-based risk prediction models for secondary prevention of cardiovascular disease: the LIPID study | the event at the baseline, modeled for secondary prevention |
|  | Short-term predictive ability of selected cardiovascular risk prediction models in a rural Bangladeshi population: a case-cohort study | compare sensitivity, sensitivity, positive and negative predictive value |
|  | A Novel Risk Score to the Prediction of 10-year Risk for Coronary Artery Disease Among the Elderly in Beijing Based on Competing Risk Model | mix laboratory and non-laboratory predictors |
|  | Predictive Accuracy of a Polygenic Risk Score–Enhanced Prediction Model vs a Clinical Risk Score for Coronary Artery Disease | laboratory-based and gene-based |
|  | A general cardiovascular risk profile: The Framingham study | laboratory-based only |
|  | Validation of the pooled cohort risk score in an Asian population - a retrospective cohort study | laboratory-based only |
|  | Who Needs Laboratories and Who Needs Statins? Comparative and Cost-Effectiveness Analyses of Non–Non-Non-Laboratory-Based, Laboratory-Based, and Staged Primary Cardiovascular Disease Screening Guidelines | effectiveness study |
|  | Predicting cardiovascular risk in England and Wales: prospective derivation and validation of QRISK2 | laboratory-based only |
|  | Derivation and validation of QRISK, a new cardiovascular disease risk score for the United Kingdom: prospective open cohort study | laboratory-based only |
|  | An independent external validation and evaluation of QRISK cardiovascular risk prediction: a prospective open cohort study | laboratory-based only |
|  | Development and validation of QRISK3 risk prediction algorithms to estimate future risk of cardiovascular disease: prospective cohort study | laboratory-based only |
|  | Predicting the impact of population-level risk reduction in cardiovascular disease and stroke on acute hospital admission rates over 5 years—a pilot study | laboratory-based only |
|  | Prediction of Coronary Heart Disease Using Risk Factor Categories | laboratory-based model development |
|  | Recalibration and validation of the SCORE risk chart in the Australian population: the AusSCORE chart | validated after recalibration |
|  | A consultation-based method is equal to SCORE and an extensive laboratory-based method in predicting the risk of future cardiovascular disease | no external validation |
|  | Assessing 10-Year Cardiovascular Disease Risk in Malaysians With Type 2 Diabetes Mellitus: Framingham Cardiovascular Versus United Kingdom Prospective Diabetes Study Equations | laboratory-based only |
|  | Predictive accuracy of the Framingham coronary risk score in British men | laboratory-based only |
|  | The QRISK was less likely to overestimate cardiovascular risk than the Framingham or ASSIGN equations | laboratory-based only |
|  | Cardiovascular/stroke risk predictive calculators: a comparison between statistical and machine learning models | laboratory-based only |
|  | Performance of the QRISK cardiovascular risk prediction algorithm in an independent UK sample of patients from general practice: a validation study | laboratory-based only |
|  | Independent external validation of cardiovascular disease mortality in women utilizing Framingham and SCORE risk models: a mortality follow-up study | laboratory-based only |
|  | Recalibrating the Non‑Communicable Diseases risk prediction tools for the rural population of Western India | recalibration study |
|  | An independent and external validation of QRISK2 cardiovascular disease risk score | laboratory-based only |
|  | Cardiovascular risk: Associated factors, assessment and agreement between WHO/ISH risk prediction chart and Framingham Scoring system among primary care patients in Kelantan, Malaysia | predictors focus |
|  | Framingham Risk Score for Prediction of Cardiovascular Diseases: A Population-Based Study from Southern Europe | compare laboratory-based only |
|  | Predictive accuracy of the Framingham general CVD algorithm in a Middle Eastern population: Tehran lipid and glucose study | laboratory-based only |
|  | Validation of continuous clinical indices of cardio-metabolic risk in a cohort of Australian adults | laboratory-markers |
|  | Copyright 2014 American Medical Association. All rights reserved. Further Insight into the Cardiovascular Risk Calculator the Roles of Statins, Revascularizations, and Under Ascertainment in the Women’s Health Study | Laboratory-based only |
|  | Comparison of 3 risk estimators to guide initiation of statin therapy for primary prevention of cardiovascular disease | laboratory-based only |
|  | Validation of the Pooled Cohort equations in a long-term cohort study of Hong Kong Chinese | laboratory-based only |
|  | Calibration and discrimination of the Framingham Risk Score and the Pooled Cohort Equations | laboratory-based only |
|  | Comparison of validation and application on various cardiovascular disease mortality risk prediction models in Chinese rural population | no external validation |
|  | Validation of Risk Prediction Models for Atherosclerotic Cardiovascular Disease in a Prospective Korean Community-Based Cohort | laboratory-based only |
|  | External validation of three atherosclerotic cardiovascular disease risk equations in rural areas of Xinjiang, China | laboratory-based only |
|  | Re-estimation improved the performance of two Framingham cardiovascular risk equations and the pooled cohort equations: A nationwide registry analysis | laboratory-based only |
|  | Predicting lifetime risk for developing atherosclerotic cardiovascular disease in Chinese population: the China-PAR project | laboratory-based only |
|  | 10-Year Coronary Heart Disease Risk Prediction Using Coronary Artery Calcium and Traditional Risk Factors: Derivation in the MESA (Multi-Ethnic Study of Atherosclerosis) With Validation in the HNR (Heinz Nixdorf Recall) Study and the DHS (Dallas Heart Study) | laboratory-based only |
|  | Derivation of a Coronary Age Calculator Using Traditional Risk Factors and Coronary Artery Calcium: The Multi-Ethnic Study of Atherosclerosis | coronary age focus |
|  | Ten-Year Coronary Heart Disease Risk Prediction Using Coronary Artery Calcium and Traditional Risk Factors: Derivation in the Multi-Ethnic Study of Atherosclerosis with Validation in the Heinz Nixdorf Recall Study and the Dallas Heart | the alternative model requires a laboratory test |
|  | Comparison of Novel Risk Markers for Improvement in Cardiovascular Risk Assessment in Intermediate-Risk Individuals | the alternative model requires a laboratory test |
|  | Gaziano TA, Pandya A, Steyn K, Levitt N, Mollentze W, Joubert G, et al. Comparative assessment of absolute cardiovascular disease risk characterization from non-laboratory-based risk assessment in South African populations | report correlation/not prospective validation |
|  | Comparison of Nonblood-Based and Blood-Based Total CV Risk Scores in Global Populations | report correlation/not prospective validation |
|  | Pandya A, Weinstein MC, Gaziano TA. A Comparative Assessment of Non-Laboratory-Based versus Commonly Used Laboratory-Based Cardiovascular Disease Risk Scores in the NHANES III Population. | report correlation/not prospective validation |
|  | Cost Effectiveness Of Non-Laboratory CVD Screening In Uzbekistan | report correlation/not prospective validation |
|  | Agreement between the laboratory-based and non-laboratory-based WHO cardiovascular risk charts: a cross-sectional analysis of a national health survey in Peru | report kappa / not prospective validation |
|  | Agreement between laboratory-based and non-laboratory-based Framingham risk score in Southern Iran | report kappa / not prospective validation |
|  | WHO Non-Lab-Based CVD Risk Assessment: A Reliable Measure in a North Indian Population. | report kappa / not prospective validation |
|  | Cardiovascular disease risk prediction in sub-Saharan African populations - Comparative analysis of risk algorithms in the RODAM study. | report kappa / not prospective validation |
|  | Comparison of laboratory-based and non-laboratory-based WHO cardiovascular disease risk charts: a population-based study | report kappa / not prospective validation |
|  | Pars cohort study of non-communicable diseases in Iran: protocol and preliminary results. International Journal of Public Health. | report kappa / not prospective validation |
|  | African partnerships through the H3Africa Consortium bring a genomic dimension to longitudinal population studies on the continent | report kappa / not prospective validation |
|  | Persistent Immune Activation and Carotid Atherosclerosis in HIV-Infected Ugandans Receiving Antiretroviral Therapy | report kappa / not prospective validation |
|  | Performance of WHO updated cardiovascular disease risk prediction charts in a low-resource setting – Findings from a community-based survey in Puducherry, India | report kappa / not prospective validation |
|  | Prevalence of cardiovascular risk factors by HIV status in a population-based cohort in South Central Uganda: a cross-sectional survey | report kappa / not prospective validation |
|  | The Atherosclerosis Risk in Communities (ARIC) Study: design and objectives. The ARIC investigators | report kappa / not prospective validation |
|  | Addressing geographical variation in the progression of non-communicable diseases in Peru: the CRONICAS cohort study protocol | report kappa / not prospective validation |
|  | Framingham Ten-Year General Cardiovascular Disease Risk: Agreement between BMI-Based and Cholesterol-Based Estimates in a South Asian Convenience Sample. | report kappa / not prospective validation |
|  | The Comparability of Lipid-based and Body Mass Index-based Cardiovascular Disease Risk Scores: Using the Rwanda 2012-2013 Non-communicable Diseases Risk Factors Survey Data | report kappa / not prospective validation |
|  | Estimation of cardiovascular risk in a rural population of Lucknow district using WHO/ISH risk prediction charts. | report kappa / not prospective validation |
|  | Application of two versions of the WHO/International Society of Hypertension Absolute Cardiovascular Risk Assessment Tools in a Rural Bangladeshi Population | report kappa / not prospective validation |
|  | Total cardiovascular risk assessment and management using two prediction tools, with and without blood cholesterol | report kappa / not prospective validation |
|  | Estimating the burden of cardiovascular risk in community dwellers over 40 years old in South Africa, Kenya, Burkina Faso, and Ghana. | report kappa / not prospective validation |
|  | Using body mass index data in the electronic health record to calculate cardiovascular risk. | report kappa / not prospective validation |
|  | Validation of the World Health Organization/ International Society of Hypertension (WHO/ISH) cardiovascular risk predictions in Sri Lankans based on findings from a prospective cohort study | report kappa / not prospective validation |
|  | Lifestyle Change, Nutrition Transition and Cardiovascular Risk in Settat Region, Morocco | focus on risk estimates |
|  | Healthy lifestyle, lipoprotein (a) levels, and the risk of coronary artery disease | focus on laboratory-based covariates |
|  | Derivation of a Protein Risk Score for Cardiovascular Disease Among a Multiracial and Multiethnic HIV+ Cohort | laboratory-based only |
|  | Cardiovascular Risk Prediction with cardio-ankle Vascular Index in the Malaysian Cohort Study | no comparison |
|  | Application of deep neural survival networks to the development of risk prediction models for diabetes mellitus, hypertension, and dyslipidemia | laboratory-based only |
|  | Recalibration of Framingham risk for a local population of Sri Lanka | adjusting the laboratory-based only |
|  | Cost-effectiveness of home-based screening of the general population for albuminuria to prevent progression of cardiovascular and kidney disease | laboratory-based- effectiveness only |
|  | Performance of the pooled cohort equations in cancer survivors: the Atherosclerosis Risk in Communities study | Laboratory-based only |
|  | Adapting cardiovascular risk prediction models to different populations: the need for recalibration | laboratory-based only |
|  | Estimated Lifetime Cardiovascular, Kidney, and Mortality Benefits of Combination Treatment with SGLT2 Inhibitors, GLP-1 Receptor Agonists, and Nonsteroidal MRA Compared With Conventional Care in Patients With Type 2 Diabetes and Albuminuria | Interventions using laboratory-based only |
|  | Recommendations for statin management in primary prevention: disparities among international risk scores | compare interventions using laboratory-based only |
|  | LDL cholesterol target attainment in cardiovascular high- and very-high-risk patients with statin intolerance: a simulation stud | Interventions assessment |
|  | Impact of lifestyle-based interventions on absolute cardiovascular disease risk: a systematic review and meta-analysis | compare interventions using laboratory-based only |
|  | Development and validation of a multicenter study on novel Artificial Intelligence-based Cardiovascular Risk Score (AICVD) | compare among laboratory-based only |
|  | Framingham risk score based vascular outcomes in acute versus chronic HIV cohorts after 6 years of ART | Laboratory-based and immunity markers |
|  | Evaluating the performance of a novel anthropometric index: weight adjusted for waist-to-height ratio (W-WHR) – for predicting cardiometabolic risk among adults in Addis Ababa | focus on predictor comparison |
|  | Comparison of the performance of cardiovascular risk prediction tools in rural India: The Rishi Valley Prospective Cohort Study | Focus on five years of CVD risk |
|  | Comparison of Laboratory and Non-Laboratory-Based 2019 World Health Organization Cardiovascular Risk Charts in the Bhutanese Population | discrimination and calibration of the model not compared |
|  | Derivation and Internal Validation of a Disease-Specific Cardiovascular Risk Prediction Model for Patients with Psoriatic Arthritis and Psoriasis | traditional factors but not compared with non-laboratory-based |
|  | BMI-based obesity classification misses children and adolescents with raised cardiometabolic risk due to increased adiposity | not modelled CVD risk |
|  | Prediction models for cardiovascular disease risk among people living with HIV: A systematic review and meta-analysis | laboratory-based only |
|  | A prediction model for left ventricular thrombus persistence/recurrence: based on a prospective study and a retrospective study | secondary CVD risk |
|  | Temporal relationships between BMI and obesity-related predictors of cardiometabolic and breast cancer risk in a longitudinal cohort | not compare model performance |
|  | Ten-Year Cardiovascular Disease Risk Score and Cognitive Function Among Older Adults: The National Health and Nutrition Examination Survey 2011 to 2014 | focus on predictors |
|  | Development and validation of a prediction model based on machine learning algorithms for predicting the risk of heart failure in middle‐aged and older US people with prediabetes or diabetes | not compared with non-laboratory-based |
|  | Comparison of LASSO and random forest models for predicting the risk of premature coronary artery disease | laboratory-based factors |
|  | Development of new scores for atherosclerotic cardiovascular disease using specific medical examination items: the Suita Study | laboratory-based only |
|  | Cardiovascular Risk Management in Persons with Dementia | focus on management |
|  | 30-Year High Cardiovascular Risk Incidence and its Determinants: CUME Study | no model comparison |
|  | Blood pressure and 10-year all-cause mortality: Findings from the PERU MIGRANT Study | predictor focused |
|  | External validation of a cardiovascular risk model for Omani patients with type 2 diabetes mellitus: a retrospective cohort study | not compared with non-laboratory-based separately |
|  | Artificial intelligence modeling to assess the risk of cardiovascular disease in oncology patients | laboratory-based only |
|  | Development of a Cardiovascular Disease Risk Prediction Model: A Preliminary Retrospective Cohort Study of a Patient Sample in Saudi Arabia | laboratory-based only |
|  | Flexible addition of risk modifiers on top of SCORE2 to improve long-term risk prediction in healthy individuals | not compared with model performance / theoretical background |
